# An MD-2-related lipid-recognition protein is required for insect reproduction and integument development

**DOI:** 10.1098/rsob.210170

**Published:** 2021-12-15

**Authors:** Wei Wang, Ya Ma, Rui-Rui Yang, Xu Cheng, Hai-Jian Huang, Chuan-Xi Zhang, Yan-Yuan Bao

**Affiliations:** ^1^ State Key Laboratory of Rice Biology and Ministry of Agriculture Key Lab of Molecular Biology of Crop Pathogens and Insect Pests, Institute of Insect Sciences, Zhejiang University, Hangzhou 310058, People's Republic of China; ^2^ State Key Laboratory for Managing Biotic and Chemical Threats to the Quality and Safety of Agro-products, Key Laboratory of Biotechnology in Plant Protection of Ministry of Agriculture and Zhejiang Province, Institute of Plant Virology, Ningbo University, Ningbo, People's Republic of China

**Keywords:** MD-2-related lipid-recognition protein, lipid metabolism, moulting, ovulation and hatching, integument, *Nilaparvata lugens*

## Abstract

The myeloid differentiation factor 2 (MD-2)-related lipid-recognition protein is involved in immune responses through recognizing bacteria lipopolysaccharide in mammals, arthropods and plants. However, the physiological roles of MD-2 in other biological processes are largely unknown. Here, we identified three homologue MD-2 genes (*NlML1*, *NlML2* and *NlML3*) by searching the genome and transcriptome databases of the brown planthopper *Nilaparvata lugens*, a hemipteran insect species. Temporospatial analysis showed that the *NlML1* gene was highly expressed in the fat body but much less so in the other tissues, while the *NlML2* and *NlML3* genes were highly expressed in the testis or digestive tract. RNA interference-mediated depletion of the *NlML1* gene significantly downregulated the transcription of numerous integument protein genes. The *NlML1* knockdown led to moulting failure and mortality at the nymph–adult transition phase, impaired egg laying and hatching, and reduced 20-hydroxyecdysone (20E) production in the nymphs. 20E could rescue the deficient moulting phenotypes derived from ds*NlML1* RNAi. These novel findings indicate that *NlML1* is required for nymphal moulting and female reproductive success as it plays an important role in regulating 20E synthesis, lipid and chitin metabolisms in *N. lugens*, thus contributing to our understanding of developmental and reproductive mechanisms in insects.

## Graphical abstract

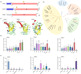

## 1. Introduction

The myeloid differentiation factor 2 (MD-2)-related lipid-recognition protein was first identified in human kidney cell lines as an extracellular binding partner of Toll-like receptor 4 (TLR4), a mammalian homologue of *Drosophila* Toll [1]. MD-2 and TLR4 form a cell surface receptor complex, which recognizes and binds to lipopolysaccharide (LPS) on the outer membrane of Gram-negative bacteria via lipid A (a glucosamine-based phospholipid that anchors LPS to the bacterial membrane to trigger immune responses) [1–3]. *In vivo* studies have demonstrated that MD-2- or TLR4-deficient mice are hyporesponsive to LPS, which provided further evidence that the TLR4/MD-2 receptor complex is essential for LPS recognition and LPS signalling [4,5]. The crystal structure of human MD-2 has been resolved in its complex with TLR4. Human MD-2 consists of two antiparallel β-sheets stabilized by three disulfide bonds, which form a hydrophilic pocket for the binding of the LPS ligand [6].

The MD-2 proteins of various organisms are known to be secreted glycoproteins that comprise an N-terminal signal peptide and a single ML (MD-2-related lipid recognition) domain of approximately 150 amino acids [7,8]. They contain at least six conserved cysteine residues, which contribute to the structure of the inter- or intra-chain disulfide bridges, and determine the formation of MD-2 dimers [9,10]. The activity of all cysteine residues is required in the interaction between MD-2 and TLR4 [10]. To date, the biological functions of MD-2 proteins have been documented in humans, mice, *Arabidopsis*, shrimps, and insects of the Diptera order (such as the mosquitoes *Aedes aegypti* and *Anopheles gambiae*, and the fruit fly *Drosophila melanogaster*) and of the Lepidoptera order (such as the silkworm *Bombyx mori* and the tobacco hornworm *Maduca sexta*) [1,2,4,11–20]. Studies on these species have revealed that MD-2 proteins play important roles in host–pathogen interactions. In humans, mice and the pacific white shrimp *Litopenaeus vannamei*, as well as in several other insect species, MD-2 proteins are key accessory proteins that mediate the host's immune defence responses against bacterial infection through LPS signalling [13]. In addition to their role in antibacterial immune functions, studies have further argued the importance of MD-2 proteins in host–virus interactions. For example, *A. aegypti* MD-2 was found to function as a dengue virus agonist and facilitate virus infection in the mosquito host [15]. A recent study reported that MD-2 proteins in kuruma shrimp *Marsupenaeus japonicus* are involved in resistance mechanisms against white spot syndrome virus [2]. Since the dengue and white spot syndrome viruses are enveloped and their outer shells are lipid-based, MD-2 proteins are thought to act as immune recognition molecules that bind to the viral lipid components during host–virus interactions. In plants, the MD-2 of *Arabidopsis thaliana* (known as *ML3*) was shown to be involved in the defence against herbivore attacks and microbial pathogens [16–21]. At present, it is widely recognized that MD-2 proteins confer molecular specificity for LPS interaction in TLR4/MD-2 complexes during immune recognition processes. However, the systematic assessment of the *in vivo* physiological functions of this type of lipid-recognition protein has not been sufficiently studied.

In this study, an important hemimetabolous rice pest, the brown planthopper *Nilaparvata lugens* (Hemiptera: Delphacidae), was used as a model insect to investigate the physiological functions of MD-2 proteins, as the whole genome sequence of this species has been previously elucidated and is susceptible to RNA interference (RNAi) [22,23]. Three *MD-2* homologous genes were identified by searching the *N. lugens* genomic and transcriptomic databases; they all contained an ML domain and were named as *NlML1*, *NlML2* and *NlML3*. Subsequently, a phylogenetic tree was constructed, which showed a diverged evolutional relationship between these three MLs. It is known that the *MD-2* homologous genes in various organisms are highly expressed in the fat bodies and haemocytes. Interestingly, in the present study it was observed that ML genes in *N. lugens* displayed different expression patterns: *NlML1* was highly expressed in the fat bodies and the eggs of adult females, while *NlML2* and *NlML3* were highly expressed in the testis or gut, but much less so in the fat bodies and the eggs. Despite containing a characteristic ML domain that is linked to the lipid-recognition function, the distinct expression patterns of these *ML* genes suggested the presence of different physiological functions. Lipids, mostly triglycerides, comprise approximately 30–40% of the oocyte dry weight and more than 50% of the fat body dry weight in insects [24]. Triglycerides are stored in lipid droplets in oocytes and fat body cells, and serve as the main energy source for oocyte maturation and embryonic development [25–27]. We recently reported that abnormalities of lipid metabolism in the fat bodies of *N. lugens* affected the storage of triglycerides in the ovaries, leading to impaired triglyceride utilization in the oocytes and failed oocyte development [28]. This study focused on the functional analysis of MLs in *N. lugens* using RNAi. Knockdown of the *NlML2* and *NlML3* genes did not cause any abnormal phenotypes. However, the knockdown of *NlML1* generated phenotypes that were apparently lethal and caused developmental arrest during the 5th instar nymphal stage, due to moulting failure. Moreover, the silencing of this gene inhibited oviposition and significantly reduced the number of eggs and hatching rates. The *NlML1-*regulated gene network was established based on a joint analysis of the transcriptome and lipid metabolome, and the results revealed that *NlML1* modulated the genetic expression of numerous lipid- and chitin-metabolic enzymes. The present study provides insights into the physiological functions of ML-containing proteins and contributes to increasing our understanding of development and reproduction mechanisms in insects.

## 2. Results

### 2.1. Bioinformatics analysis and temporospatial expression patterns of *Nilaparvata lugens ML* genes

**Figure 1.**
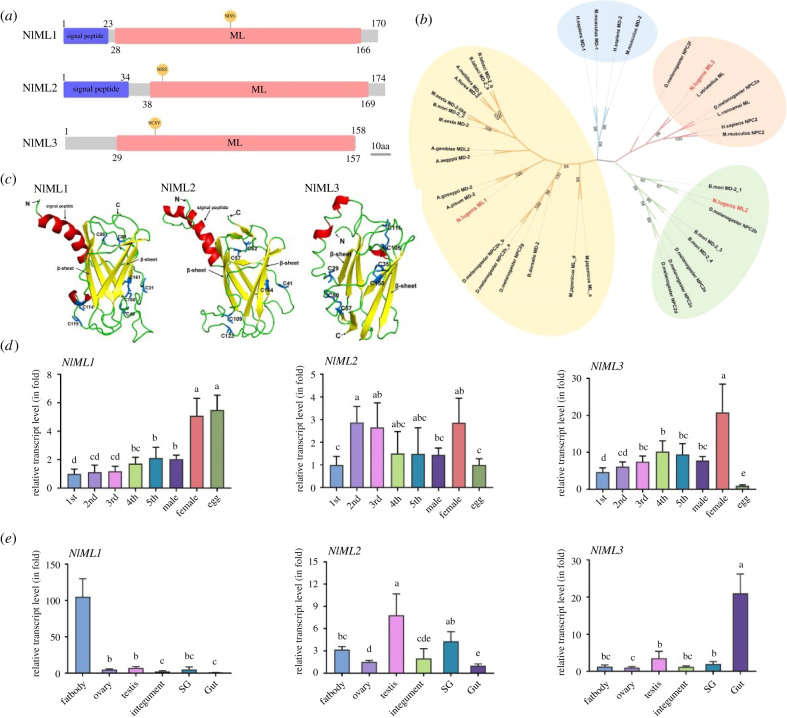
Bioinformatics analysis and temporospatial expression patterns of *N. lugens ML* genes. (*a*) Prediction of the domain structures of NlML proteins. Conserved domains in the deduced amino acid sequences of *NlML*s were determined using SMART, Pfam and NCBI. The putative signal peptides and ML domains are shown by blue and red bars, respectively. The NISS and NCSV amino acids refer to the predicted glycosylation sites. Grey bars represent the number of amino acid residues. (*b*) Phylogenetic analysis of NlMLs with the homologous ML proteins. The phylogenetic tree was constructed based on ML protein sequences from human, mouse, shrimp and insect species using the maximum-likelihood method in Mega X (http://www.megasoftware.net/). Phylogenetic relationships were determined using the Jones–Taylor–Thornton model for amino acid substitution. Bootstrap analysis was set for values of 1000. (*c*) Three-dimensional model structures of NlMLs, constructed using Phyre2 (http://www.sbg.bio.ic.ac.uk/~phyre2) and PyMOL software. N and C represent the N- and C-terminus of the proteins. The signal peptide and β-sheet are shown in red and yellow, respectively. (*d*) Developmental stage-specific expression analysis (in fold) of *NlML*s. Total RNAs were extracted from mixed eggs at different time points after laying (1 h, *n* = 20; 24 h, *n* = 20; 48 h, *n* = 20; 72 h, *n* = 20; 96 h, *n* = 20; 120 h, *n* = 20; 144 h, *n* = 20; 168 h, *n* = 20), first-instar (*n* = 100), second-instar (*n* = 50), third-instar (*n* = 50), fourth-instar (*n* = 30) and fifth-instar nymphs (*n* = 20), and male (*n* = 20) and female adults (*n* = 20). The numbers 1st, 2nd, 3rd, 4th and 5th refer to 1st–5th instar nymphs. (*e*) Tissue-specific expression analysis (in fold) of *NlMLs.* Total RNAs were extracted from fat body (*n* = 50), ovary (*n* = 30), integument (*n* = 50), salivary gland (*n* = 100) and gut (*n* = 50) were dissected from female adults, and testis (*n* = 50). Relative transcript levels of the target genes in each tissue were determined by qRT-PCR as described above. The relative transcript levels of each gene in each sample were normalized using the *N. lugens β*-*actin* threshold cycle (*C*_t_) values obtained from reactions run on the same plate. In each assay, the transcript level was normalized to the lowest level, which was arbitrarily set at one. The statistical analysis was performed using a one-way ANOVA followed by a Games–Howell *post hoc* test (*p* < 0.05) with SPSS 18.0 (Chicago, Illinois, USA). The data were plotted with GraphPad Prism v. 8 (San Diego, CA, USA). Three independent biological replicates (mean ± s.d.) were conducted and relative transcript levels in each sample were measured using the 2^−ΔΔCt^ method.

Three *ML* DNA sequences were identified by searching the *N. lugens* genomic and transcriptomic databases. Their deduced amino acid sequences contained an ML domain (Pfam domain PF02221.15), and the genes were named as *NlML1*, *NlML2* and *NlML3*. The cDNA sequences of the *NlML1* and *NlML2* genes had open reading frames of 513 and 525 nucleotides encoding 170 and 174 amino acid residues with the theoretical molecular masses of 19.3 and 19.4 kDa, respectively. The deduced proteins had predicted secretory signal peptides of 23 and 34 amino acids at their N-terminus (figure 1*a*). The cDNA sequence of the *NlML3* gene had an open reading frame of 477 nucleotides encoding 158 amino acid residues with the theoretical molecular mass of 17.4 kDa. Its deduced protein lacked a predicted N-terminal signal peptide sequence, suggesting it is a non-secretory protein. The ML domains nearly spanned the entire coding regions of the NlML1 (amino acids 28–166), NlML2 (amino acids 38–169) and NlML3 (amino acids 29–157) proteins (figure 1*a*). Despite being a characteristic ML domain, the percentage of sequence similarities that these three MLs shared at the amino acid level were low; specifically, 20% between NlML1 and NlML2, 13.1% between NlML1 and NlML3 and 13.7% between NlML2 and NlML3. NlML1 showed significant similarities with the MD-2 proteins of several insect species belonging to the Lepidoptera, Diptera, Hemiptera and Hymenoptera orders (electronic supplementary material, figure S1). All of these ML domain proteins detected in insect species contain eight conserved cysteine residues, as opposed to the mammalian homologues that contain only six. To understand the evolutionary relationships, a phylogenetic tree was constructed using ML domain proteins from humans, mice, shrimps and insects, based on their current annotation. The MLs of *N. lugens* diverged into three independent branches, while the MD-1 and MD-2 proteins of humans and mice formed a single independent branch (figure 1*b*). NlML1 was closely related to the MD-2 and Niemann–Pick type C2 (NPC2) proteins of insect species—among which was the fruit fly *D. melanogaster* (NPC2 h-b, NPC2 h-a, NPC2 g)—and to the MLs of shrimps. NlML2 clustered with the MD-2 proteins of the silkworm *B. mori* and with the NPC2b, NPC2c, NPC2d and NPC2e proteins of *D. melanogaster*. NlML3 was the most closely related to the ML domain proteins of the insect *Laodelphax striatellus* (Hemiptera), and had close relationships with the NPC2f and NPC2a proteins of *D. melanogaster*, a shrimp ML and mammalian NPC2 proteins. The three-dimensional model structures of *N. lugens* ML proteins were built using PyMOL (electronic supplementary material). Three MLs showed similar overall structures with identical β-strand organization consisting of 8–10 β-strands that formed two antiparallel β-sheets and a large hydrophobic cavity between the two β-sheets (figure 1*c*). However, the structures indicated the presence of substantial differences between certain regions, and the most pronounced structural deviations were found in the signal peptide regions. Both NlML1 and NlML2 contained N-terminal α-helix signal peptides, while NlML3 presented no signal peptide. In addition, a significant variation was found in the cysteine residues: NlML1, NlML2 and NlML3 contained eight, six and seven cysteines, respectively. NlML3 was predicted to have three intramolecular disulfide bridges with the distances of 2.1 Å resolution for C35–C150, 2.0 Å for C50–C57 and 2.1 Å for C105–C111. For NlML1 and NlML2, as the nearest bridge distances connecting each two cysteines were greater than 2.4 Å, the intramolecular disulfide bonds could not be accurately predicted. We investigated the expression patterns of *NlML* genes throughout each developmental stage and in different tissues by qRT-PCR analysis using a relative quantitative method. *NlML1* transcripts were detected at high levels in eggs (mixed eggs from different time points after laying) and female adults, but at low levels in 1st–5th instar nymphs and male adults (figure 1*d*). *NlML2* transcript levels were significantly higher in female adults compared with eggs and 1st instar nymphs. There were no significant differences between female adults and 2nd–5th instar nymphs. *NlML3* showed significantly high transcript levels in female adults and extremely low levels in eggs compared with 1st–5th instar nymphs and male adults. Tissue-specific expression patterns were then determined in adults, and qRT-PCR analysis showed that *NlML1* transcripts were detected at significantly higher levels in the fat bodies than in the other tested tissues including ovaries, testes, integuments, salivary glands and guts (figure 1*e*). *NlML2* transcripts were detected at significantly higher levels in the testes compared with the fat bodies, ovaries, integuments and guts, while *NlML3* transcripts were detected at the highest levels in guts among the tested tissues, suggesting the possibility that these MLs have different functional roles. Expression of *NlML1* transcripts in relation to developmental stage showed relatively high levels in mixed eggs, while tissue-specific expression showed relatively low levels in ovaries. We therefore compared *NlML1* gene expression levels in ovaries and eggs at different time points after laying. qRT-PCR analysis indicated that *NlML1* transcript levels were relatively low in female ovaries containing oocytes and in newly laid eggs 1 h after laying, but then increased quickly to relatively high levels at 24 h and gradually decreased again at 48–168 h after laying (electronic supplementary material, figure S2).

### 2.2. Functional analysis of *Nilaparvata lugens ML* genes via RNAi

**Figure 2.**
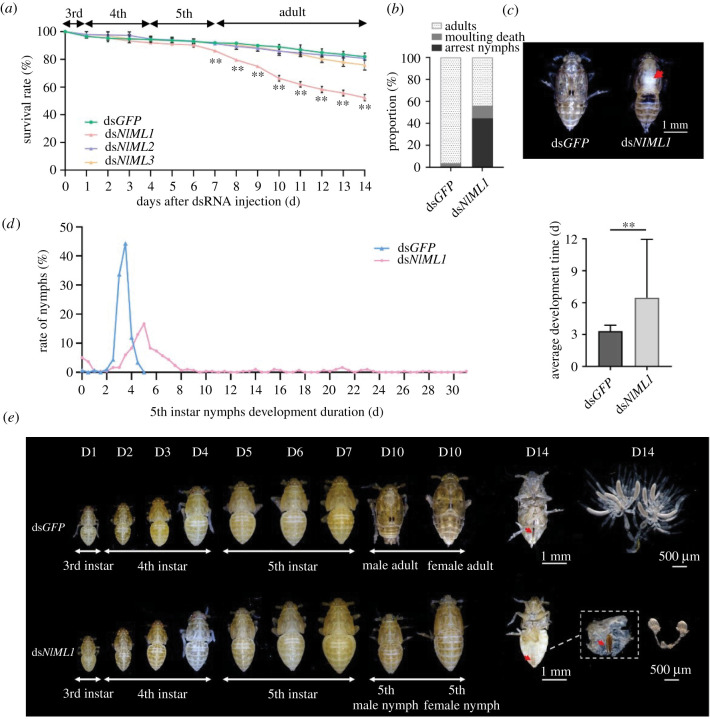
Functional analysis of *N. lugens ML* genes via RNAi. (*a*) Dynamic analysis of *N. lugens* survival rates. Each 3rd instar nymph was injected with ds*NlML* and phenotypic variations were observed at 24 h intervals. Ds*GFP* was injected as a negative control to determine the non-specific effects of dsRNA. For each treatment, the survival rates were calculated from three independent biological replicates (mean ± s.d.; *n* = 100 insects). (*b*) Proportion of different phenotypes in the nymph–adult moulting transition after *NlML1* knockdown. The 3rd instar nymphs were injected with ds*NlML1* and ds*GFP*. Phenotypes were observed in the 5th instar nymphs and adults. The term ‘adults’ refers to the nymphs that completed the moulting transition from 5th instar stage to adults; ‘moulting death’ refers to the nymphs that suffered lethal deficits at the nymph–adult transition; ‘arrested nymphs’ refers to the nymphs that underwent developmental arrest at the 5th instar stage. (*c*) Observations of the deficient moulting phenotype. The 3rd instar nymphs were injected with ds*NlML1* and the phenotypes at the nymph–adult moulting transition were observed. Arrowhead indicates the split dorsal ecdysial line. (*d*) Calculation of development duration in the 5th instar nymphs. The 3rd instar nymphs were injected with ds*NlML1* or ds*GFP* and the 5th instar nymphal development duration was recorded and calculated at 12 h intervals. Each nymph was set as an independent biological replicate, with 300 independent biological replicates for each treatment (mean ± s.d.; *n* = 300 insects). The average development time at the 5th instar stage is shown in the right graphic. (*e*) Morphological observations of *N. lugens* developmental stages. The 3rd instar nymphs were injected with ds*NlML1* or ds*GFP*. Morphology was observed at each developmental stage after RNAi. The ds*GFP*-injected adult females showed a fully developed ovipositor and fully developed ovaries at 14 dpi. The ds*NlML1*-injected female nymphs showed a fully developed ovipositor and non-developed ovaries at 14 dpi. Arrowheads indicate the ovipositors.

To understand the physiological functions of *ML* genes, the transcript levels of three *ML* genes were knocked down through an *in vivo* RNAi approach applied to *N. lugens* nymphs. Knockdown of the *NlML1* transcription generated an apparently lethal phenotype. Following RNAi in the 3rd instar nymphs, greater than 90% of ds*NlML1*-injected individuals completed the moulting transitions of 3rd–4th instar and 4th–5th instar nymphs, and survived 6 days post-injection (dpi). However, the survival rates significantly decreased at the transition of 5th instar nymphs into adults at 7 dpi and declined to less than 60% at 14 dpi (figure 2*a*). Injection of ds*NlML2* or ds*NlML3* had no significant difference with ds*GFP* injection on the survival of *N. lugens* throughout the transitioning of 3rd instar nymphs into adults; in these cases, the survival rates were more than 80% at 14 dpi (figure 2*a*). Based on the RNAi results, the *NlML1* gene was examined in order to elucidate its functional roles during developmental processes. It was observed that more than 95% of ds*GFP*-injected nymphs successfully completed the moulting transition from 5th instar nymphs to adults (figure 2*b*). By contrast, only 44% of the ds*NlML1*-injected nymphs reached the adult stage (figure 2*b*). In particular, 45% of the nymphs underwent developmental arrest at the 5th instar stage, while another 11% had lethal deficits during the nymph-to-adult transition around 7–8 dpi (figure 2*b*). The lethal deficit was manifested in the old integument being split along the dorsal ecdysial line, and yet they remained attached to the abdomens of the ds*NlML1*-injected 5th instar nymphs (figure 2*c*). These nymphs were not able to complete moulting and died. Those nymphs that underwent developmental arrest at the 5th instar stage were, in large part, unable to develop into adults and they also gradually died; only a few individuals reached the adult stage. Among ds*NlML1*-injected nymphs, 38% lasted 5 ± 0.5 days at the 5th instar stage, compared with 90% of ds*GFP*-injected nymphs, which lasted 3.5 ± 0.5 days at the 5th instar stage (figure 2*d*, left panel). Surprisingly, some ds*NlML1*-injected nymphs underwent very long developmental delays of more than 10 days, reaching a record period of 31 days at the 5th instar stage (figure 2*d*, left panel). The average duration of development observed for ds*NlML1*-injected nymphs at the 5th instar stage was 6.5 days, which was significantly longer than the 3.3 days seen in the ds*GFP*-treated controls (figure 2*d*, right panel). Post-injection observations of developmental processes showed that the ds*GFP*-injected 3rd instar nymphs successfully completed the nymph–nymph transition before 5 dpi and the nymph–adult transition around 7 dpi. By contrast, 45% of ds*NlML1*-injected nymphs, which experienced developmental arrest, were not able transition into adults at or after 7 dpi following RNAi. The phenotype associated with developmental arrest in the ds*NlML1*-injected nymphs is shown in figure 2*e*. At 10 dpi, these nymphs remained arrested at the 5th instar stage, while the ds*GFP*-injected nymphs had developed into adults by that time. Morphological observations showed that the ds*GFP*-injected female adults had fully developed ovaries and ovipositor in their abdomen integuments at 14 dpi (figure 2*e*). By contrast, the arrested 5th instar female nymphs that were injected with ds*NlML1* showed very small and non-fully developed ovaries. Interestingly, they did present a fully developed ovipositor in the abdomen integuments (figure 2*e*, right panels). Generally, *N. lugens* nymphs at the end of the 5th instar stage have a non-fully developed ovipositor. qRT-PCR analysis confirmed that the transcript levels of *ML* genes were notably reduced in the ds*NlML*-injected insects compared to the levels detected in the ds*GFP*-injected controls at 3 dpi. Silencing of one of the *NlML* genes did not affect expression of the others, suggesting that there was no compensatory mechanism (electronic supplementary material, figure S3).

### 2.3. Detection of NlML1 proteins *in vivo* by immunofluorescence staining assay

**Figure 3.**
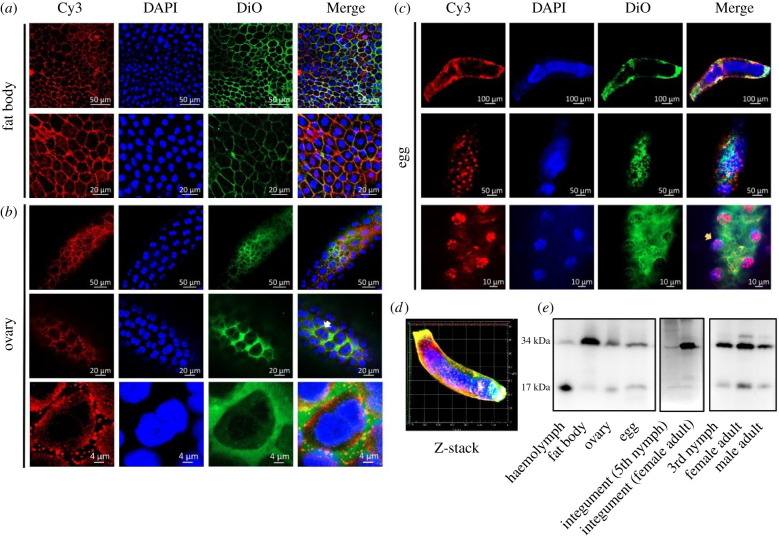
Immunofluorescence staining and western blotting assays. The location of NlML1 proteins was determined in (*a*) fat body, (*b*) ovaries and (*c*) laid eggs by immunofluorescence assay. Red fluorescence signals indicate Cy3-labelled NlML1 proteins and greenish fluorescence signals indicate Dio-labelled cell membranes. Blue fluorescence signals show nuclei stained by DAPI. (*d*) A three-dimensional image of the laid eggs obtained by Z-stack mode. (*e*) Western blotting assay showing the specific NlML1 protein bands in *N. lugens* tissues and whole bodies. Haemolymph, fat body and ovary samples were collected from female adults on the 3rd day after emergence; eggs were collected 88 h after the laying period; integuments were removed from the 5th instar nymphs and female adults. Whole-body samples were collected from the 3rd instar nymphs, female and male adults. White and yellow arrows indicate the follicular epithelial cells of the developing oocytes and the blastoderm cells of the laid eggs, respectively.

An immunofluorescence staining assay was conducted to determine the location of NlML1 proteins in the fat bodies and ovaries of female adults on the 3rd day after emergence and in the eggs at 88 h after the laying period, based on the observation that *NlML1* was highly expressed in fat bodies and eggs, as shown in figure 1. Given that the ovaries contain the developing oocytes on the 3rd day after emergence, we determined the location of NlML1 proteins in the ovaries. NlML1 proteins were clearly visualized in the cell membranes of the fat bodies (figure 3*a*). In *N. lugens*, the developing oocytes found in the ovaries were surrounded by a single layer of follicular cells (figure 3*b*). At the start of vitellogenesis, each follicular cell generates two nuclei, positioned on top of each other along the central axis of each cell. NlML1 proteins were detected in the membranes of the follicular epithelial cells and in the intercellular space (figure 3*b*). In the laid eggs, NlML1 proteins were located in the *blastoderm* cells (figure 3*c*) and shown by the three-dimensional images obtained from the Z-stack mode (figure 3*d*). A lateral view of the laid eggs clearly showed that NlML1 proteins were located outside the nuclei of the *blastoderm* cells (electronic supplementary material, figure S4). A western blotting assay detected a specific protein band around 17 kDa using an antiserum against NlML1 in the haemolymph of female adults (figure 3*e*). Weak protein bands with similar size were detected in the fat bodies, ovaries, integuments of female adults and laid eggs as well as in the whole bodies of 3rd instar nymphs and adults. The size of the specific protein band was consistent with the theoretical molecular mass (16.8 kDa) of the NlML1 protein without a signal peptide sequence. In addition, protein bands of approximately 34 kDa were also detected in the fat bodies, ovaries, integuments, laid eggs and in the whole bodies of the 3rd instar nymphs and adults (figure 3*e*). A very faint protein band of 34 kDa was observed in the haemolymph of the female adults, and as its size was almost double that of the theoretical molecular mass (16.8 kDa) it is suggested that NlML1 was present in two physical forms: a dimer in the fat body, ovary, integument and eggs; and a free monomer in the haemolymph.

### 2.4. Effects of *NlML1* knockdown on female reproduction

**Figure 4.**
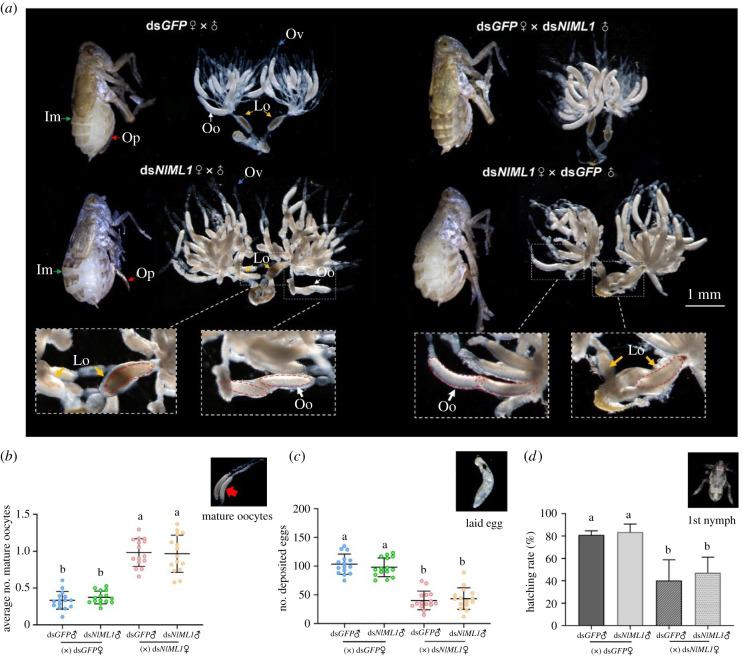
Effects of *NlML1* RNAi on female reproduction in *N. lugens*. (*a*) Morphologic observations of the bodies and ovaries of *NlML1*-injected adult females on the 7th day after emergence. Abbreviations: Im, intersegment; Op, ovipositor; Ov, ovariole; Oo, mature oocyte; Lo, lateral oviduct. Scale bar, 1.0 mm. The enlarged images show the mature oocytes located in the ovariole and the lateral oviduct is indicated by red dotted lines. (*b*) Calculation of the number of mature oocytes in the ovarioles. Adult females and males were mated on the 2nd day after emergence, and banana-shaped mature oocytes in each ovariole in each mated pair were calculated. The *x*-axis indicates each mating pair (ds*GFP*-injected female and ds*GFP*- or ds*NlML1*-injected male or ds*NlML1*-injected female and ds*GFP*- or ds*NlML1*-injected male). The *y*-axis shows the average number of mature oocytes in each ovariole. Fifteen independent biological replicates (mean ± s.d) were carried out (*n* = 15 ♀ × ♂). An example of a typical banana-shaped mature oocyte is shown in the upper right corner of the graph. (*c*) Calculation of the number of laid eggs. Mating was performed between a female and a male adult on the 2nd day after emergence. The number of eggs laid in rice leaf sheaths from each mating pair was then calculated. The *x*-axis indicates each mating pair, namely, a ds*GFP*-injected female and a ds*GFP*- or a ds*NlML1*-injected male; a ds*NlML1*-injected female and a ds*GFP*- or a ds*NlML1*-injected male. The *y*-axis shows the number of eggs laid by each mating pair. Fifteen independent biological replicates (mean ± s.d.) were carried out (*n* = 15 ♀ × ♂). An example of laid egg with an eye spot is shown in the upper right corner of the graph. (*d*) Determination of hatching rates. Hatching rates were calculated based on the eggs laid in rice leaf sheaths by each mating pair, as described above. Fifteen independent biological replicates (mean ± s.d.) were carried out (*n* = 15 ♀ × ♂). An example of newly hatched 1st instar nymph is shown in the upper right corner of the graph. The statistical analysis was performed using a one-way ANOVA followed by Tukey's *post hoc* test (*p* < 0.05).

As NlML1 proteins were located in the ovaries and eggs, their functional roles in reproductive processes were investigated through RNAi. Mating occurred between the female and the male adult on the 2nd day after emergence. The ds*NlML1*-injected female adults that mated with ds*NlML1*- or ds*GFP*-injected males showed an abnormally expanded abdomen and stretched intersegmental membranes in the tergum, whereas the ds*GFP*-injected female controls that mated with ds*NlML1*- or ds*GFP*-injected males had a flat abdomen around the 7th–10th day after emergence, which corresponds to the mid to late stages of female oviposition (figure 4*a*). To further confirm these observations, the ovaries of female adults were dissected, the number of mature oocytes was calculated and morphological changes were observed on the 7th day after emergence. The number of mature oocytes, arranged in the typical banana-shape, at the bottom of the ooecia, was greater in the ovaries of ds*NlML1*-injected females that mated with ds*NlML1*- or ds*GFP*-injected males than in the ovaries of ds*GFP*-injected females that mated with ds*NlML1*- or ds*GFP*-injected males (figure 4*a*,*b*). The average number of mature oocytes in ds*NlML1*-injected females was significantly more than that in the ds*GFP*-treated controls. Each mating between a ds*NlML1*-injected female and a male (ds*NlML1*♀ × ♂) or between a ds*NlML1*-injected female and a ds*GFP*-injected male (ds*NlML1*♀ × ds*GFP*♂) generated 0.96 and 0.98 mature oocytes in each ovariole of the ovaries, respectively, whereas each mating between a ds*GFP*-injected female and a male (ds*GFP*♀ × ♂) or between a ds*GFP*-injected female and a ds*NlML1*-injected male (ds*GFP*♀ × ds*NlML1*♂) generated 0.33 and 0.37 mature oocytes in each ovariole of the ovaries, respectively (figure 4*b*). In *N. lugens*, the female adults on the 7th day after emergence were at the mid-late stages of oviposition. A part of ovarioles contained only one mature oocyte, and the other ovarioles had no mature oocyte due to oviposition, as observed in ds*GFP*-injected female controls. However, some ovarioles in ds*NlML1*-injected females generated two or three mature oocytes. Interestingly, one to two mature oocyte(s) were located in the lateral oviduct in the ds*NlML1*-injected females, but none of these oocytes (in the ovarioles or in the lateral oviduct) could be laid in the rice leaf sheaths. Video footage indicates that the ds*NlML1*-injected females failed to lay eggs after trying to insert the ovipositors in rice leaf sheaths multiple times (electronic supplementary material, movie S1), and their ovipositors could not be retracted into their abdomen. By contrast, ds*GFP*-injected females successfully laid eggs in the leaf sheaths (electronic supplementary material, movie S2). The average number of eggs laid by ds*NlML1*-injected females was significantly lower than that laid by the ds*GFP*-treated controls. Each mating between a ds*NlML1*-injected female and a male (ds*NlML1*♀ × ♂) or between a ds*NlML1*-injected female and a ds*GFP*-injected male (ds*NlML1*♀ × ds*GFP*♂) produced 44 and 40 eggs, respectively, whereas each mating between a ds*GFP*-injected female and a male (ds*GFP*♀ × ♂), or between a ds*GFP*-injected female and a ds*NlML1*-injected male (ds*GFP*♀ × ds*NlML1*♂) generated 98 and 103 eggs, respectively (figure 4*c*). Moreover, knockdown of the *NlML1* transcription significantly affected egg hatching in rice leaf sheaths. Only 40–47% of the eggs laid after the mating between a ds*NlML1*-injected female and a ds*NlML1*- or a ds*GFP*-injected male hatched into nymphs (figure 4*d*). By contrast, 81–84% of the eggs generated from the mating of a ds*GFP*-injected female and a ds*GFP*-injected male or a ds*NlML1*-injected male successfully hatched into the nymph stage. These results clearly suggest that the knockdown of *NlML1* affects oviposition and hatching in *N. lugens*.

### 2.5. Identification of *NlML1-*regulated gene networks in *Nilaparvata lugens*

**Figure 5.**
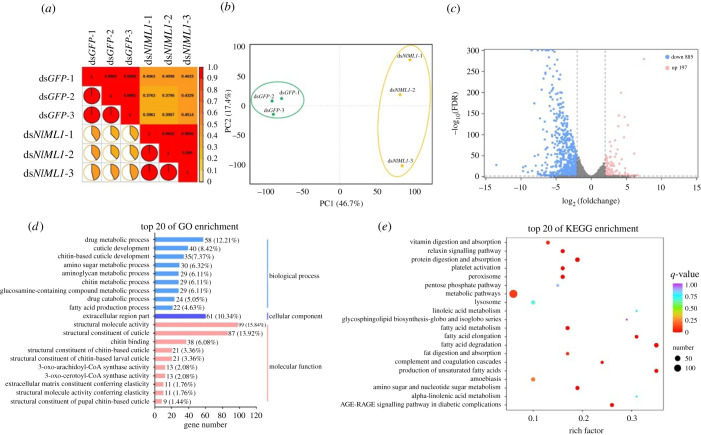
Analysis of comparative transcriptome data. (*a*) Pearson correlations between the biological replicates of ds*NlML1*- and ds*GFP*-injected samples. (*b*) Principal component (PC) analysis of expressed genes. Each dot represents an independent biological replicate. (*c*) Volcano plot of differentially expressed genes (DEGs) between ds*NlML1* and ds*GFP* treatments. Blue and red dots represent genes that were significantly downregulated and upregulated, respectively; grey dots show non-significantly different genes. Identification of the different gene expression is based on the criteria of log_2_ FC > 2 and FDR < 0.05. (*d*) Histogram representation of GO enrichment. DEGs are enriched in three main categories: biological process, cellular component and molecular function. The *x*-axis indicates the number of genes in a category and the percentage of a specific genetic pathway in all enriched DEGs. (*e*) KEGG enrichment scatter plot. The *x*-axis shows the rich factor and the *y*-axis shows the DEGs enrichment in the KEGG pathways. The sizes and colours of the dots represent the number of DEGs and their level of enrichment in the pathways.

To identify *NlML1-*regulated gene networks, a global expression profiling was conducted using RNA-Seq techniques to compare changes in gene expression in ds*NlML1*- and ds*GFP*-injected 5th instar nymphs. Pairwise correlations between variables in the RNA-seq datasets were evaluated using the Pearson correlation coefficient. The correlation coefficients (*R*^2^) between the biological replicates in ds*NlML1*- or ds*GFP*-injected groups were high (*R*^2^ > 0.989), while those between the two treatment groups were low (*R*^2^ < 0.462) (figure 5*a*), indicating an excellent level of scientific reproducibility of the experiments. Principal components analysis showed that ds*GFP*-injected and ds*NlML1*-injected samples diverged (figure 5*b*). The ds*GFP*-injected replicates clustered together in a small area and the first principal component clearly separated them from the ds*NlML1*-injected samples, which were differentiated into a separate cluster, suggesting a clear differentiation between the two treatments. Based on the criteria of log_2_ fold change (FC) greater than 2 and log_10_ fold discovery rate (FDR) values less than 0.05, 1082 differentially expressed genes (DEGs) were identified, of which 885 were significantly downregulated and 197 were significantly upregulated in the *NlML1*-injected nymphs compared with the ds*GFP*-injected controls (figure 5*c*; electronic supplementary material, table S1). These DEGs were entered into gene ontology (GO) in order to elucidate biological processes and into the Kyoto Encyclopaedia of Genes and Genomes (KEGG) to perform signalling pathway enrichment analysis. The 20 highest ranking GO terms showed that the DEGs were highly enriched in various biological processes, including drug metabolism, chitin-based cuticle development, amino sugar/aminoglycan metabolism and fatty acid production. Their molecular functions were mainly associated with structural molecular activity and integument structure (figure 5*d*; electronic supplementary material, table S2). KEGG analysis revealed that the DEGs were mainly enriched in the signalling pathways of lipid metabolism, including fatty acid elongation and degradation, production of unsaturated fatty acids and fat digestion/absorption. In addition, DEGs were enriched in human disease-related signalling pathways involving also diabetic complications and platelet activation (figure 5*e*; electronic supplementary material, table S3). Comparative transcriptomic analysis suggests that *NlML1* is closely correlated with lipid metabolism.

### 2.6. Lipid metabolomic profiling analysis

**Figure 6.**
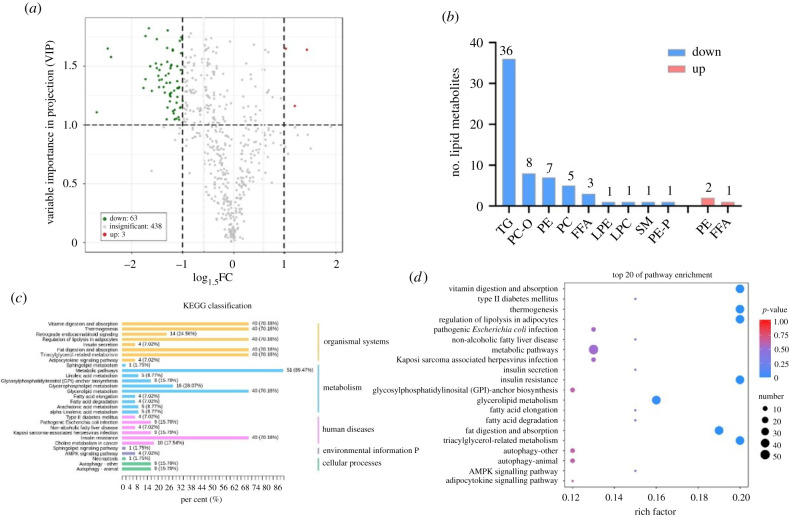
Analysis of lipid metabolomic profiling. (*a*) Volcano plot of differential lipid metabolites (DLMs) between ds*NlML1* and ds*GFP* treatments. Green and red dots represent the significantly decreased and increased lipid metabolites, respectively; grey dots show non-significantly different lipid metabolites. Screening of the DLMs is based on the criteria of variable importance in projection (VIP) ≥ 1 and |log_1.5_(FC)| ≥ 1. (*b*) Classification of the 66 differential lipid metabolites. TGs, triacylglycerols; PC-O, phosphatidylcholine-Os; PE, phosphatidylethanolamines; PC, phosphatidylcholines; FFA, free fatty acids; LPE, lysophosphatidylethanolamine; LPC, lysophosphatidylcholine; SM, sphingomyelin; PE-P, phosphatidylethanolamine-P. (*c*) Histogram representation of KEGG enrichment. The DLMs are enriched in five main categories: organismal systems, metabolism, human diseases, environmental information processing and cellular processes. The *x*-axis indicates the number of lipid species enriched in a pathway and the percentage of lipid species in that specific pathway relative to all enriched DLMs. (*d*) KEGG enrichment scatter plot. The *x*-axis shows the rich factor and the *y*-axis shows the DLMs enrichment in the KEGG pathways. The sizes and colours of the dots represent the number of DLMs and their level of enrichment in the pathways.

A lipid metabolomic profiling analysis was conducted based on the RNA-seq results. The differential lipid metabolites (DLMs) were screened between ds*NlML1*- and ds*GFP*-injected nymphs using the criteria of VIP ≥ 1 and |log_1.5_(FC)| ≥ 1. A total of 504 DLMs were detected in the metabolome, among which 66 significantly differential lipid species were identified (figure 6*a*). Of these, 63 lipid species presented notably reduced levels in the ds*NlML1*-injected group compared to the ds*GFP*-injected group, in the following nine major lipid classes: 36 triacylglycerols (TGs), eight phosphatidylcholine-Os (PC-O), seven phosphatidylethanolamines (PE), five phosphatidylcholines (PC), three free fatty acids (FFA), one lysophosphatidylethanolamine (LPE), one lysophosphatidylcholine (LPC), one sphingomyelin (SM) and one phosphatidylethanolamine-P (PE-P) (figure 6*b*; electronic supplementary material, table S4). Three lipid species, mainly enriched in PE (2) and FFA (1), were significantly enhanced in the ds*NlML1*-injected group. KEGG pathway analysis showed that the 66 DLMs were mainly enriched in 29 pathways, which are classified into five groups: organismal systems, metabolism, human diseases, environmental information processing and cellular processes (figure 6*c*). The results of the analysis revealed that nine of 20 highest ranking enrichment pathways were correlated with lipid metabolism, and they are listed as follows: regulation of lipolysis in adipocytes (KO:04923), non-alcoholic fatty liver disease (KO:04932), glycosylphosphatidylinositol (GPI)-anchor biosynthesis (KO:00563), glycerolipid metabolism (KO:00561), fatty acid elongation (KO:00062), fatty acid degradation (KO:00071), fat digestion and absorption (KO:04975), TG-related metabolism (KO:04979) and adipocytokine signalling pathway (KO:04920) (figure 6*d*; electronic supplementary material, table S5). The pathway analysis also revealed that there was a correlation with several insulin-related enrichment pathways, including type II diabetes mellitus, insulin secretion and insulin resistance. These results further suggest that the functional roles of *NlML1* are linked to lipid metabolism.

### 2.7. Joint analysis of the transcriptome and metabolome profiling

**Figure 7.**
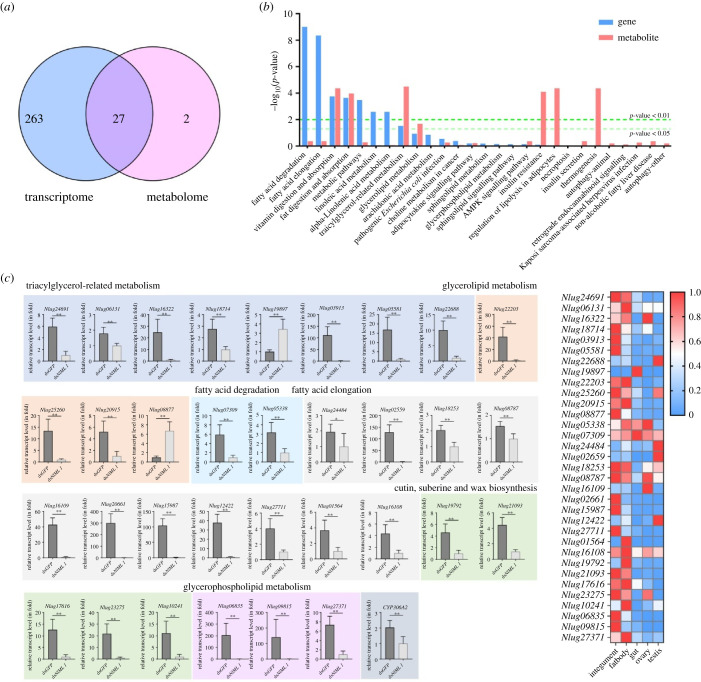
Joint analysis of the transcriptome and metabolome profiling. (*a*) Venn diagram indicating the number of DEG and DLM enrichment pathways. (*b*) Overlapping pathways of DEG and DLM enrichments. Blue and red colours represent genes and lipid metabolites enriched in each pathway, respectively. (*c*) Verification of changes in the expression of DEGs by qRT-PCR assay. RNAi was performed by microinjecting ds*NlML1* or ds*GFP* into the 3rd instar nymphs. Total RNAs were extracted from the dsRNA-injected 5th instar nymphs at 72 h. The relative transcript levels of the target genes were determined by qRT-PCR as described in figure 1. The relative transcript levels of each gene were normalized using *N. lugens 18S rRNA* threshold cycle (*C*_t_) values obtained from reactions run on the same plate. Statistical analysis was performed using GraphPad Prism v. 8 software (San Diego, CA, USA). Three independent biological replicates (mean ± s.d.) were conducted and relative transcript levels in each sample were measured using the 2^−ΔΔCt^ method. *p < 0.05, **p < 0.01 between ds*NlML1* and ds*GFP*-injected nymphs (Student's *t*-test). (*d*) Tissue-specificity analysis of DEGs. Total RNAs were extracted from the ovaries of female adults, testis of male adults and from the fat body, gut and integument of 5th instar nymphs (*n* = 50–100). The qRT-PCR assay was performed as described in figure 1. The relative transcript levels of each gene in each developmental stage were normalized using *N. lugens β*-*actin* threshold cycle (*C*_t_) values obtained from reactions run on the same plate. A heatmap was plotted using GraphPad Prism v. 8 software based on the qRT-PCR data obtained from three biological replicates. Red and blue colours refer to the high and low transcript levels of target genes. The *y*-axis shows the gene ID in the transcriptome and the *x*-axis shows the different tissues.

A joint analysis of the transcriptome and metabolome profiling revealed a correlation between DEGs and DLMs, which were enriched in 290 and 29 signalling pathways, respectively (figure 7*a*), and showed that 27 of these pathways were shared by both DEGs and DLMs. The overlapping pathways were mainly associated with lipid metabolisms including fatty acid degradation and elongation, vitamin digestion and absorption, fat digestion and absorption, linoleic acid metabolism, alpha-linolenic acid metabolism, TG-related metabolism, glycerolipid metabolism, arachidonic acid metabolism, adipocytokine signalling pathway, glycerophospholipid metabolism, sphingolipid metabolism and regulation of lipolysis in adipocytes (figure 7*b*; electronic supplementary material, table S6). There were 39 genes participating in these pathways, and their transcript variations were analysed in ds*NlML1*- and ds*GFP*-injected nymphs by qRT-PCR (figure 7*c*). The results confirmed that 33 out of 39 genes presented changes in expression that were similar to those obtained from RNA-seq (electronic supplementary material, table S7). The transcript levels of these genes were significantly modified in the nymphs after *NlML1* RNAi. These genes included: a *Niemann–Pick type C1* (*NPC1*); a *low-density lipoprotein receptor adapter protein 1* (*LDLRAP1*); four *triacylglycerol lipase* (*TGL*) genes and two *scavenger receptor class B type 1* (*SR-B1*) genes in the TG-related metabolism pathway; a *pancreatic lipase-related protein 1* (*PLRP1*) and three *pancreatic triacylglycerol lipase* (*PNLIP*) genes in the glycerolipid metabolism pathway; an *acetyl-CoA acetyltransferase* (*ACAT*) and an *acyl-CoA oxidase* (*ACOX*) gene in the fatty acid degradation pathway, a *very-long-chain of 3-oxoacyl-CoA reductase* (*VLoxCAR*), a *very-long-chain of* (*3R*)*-3-hydroxyacyl-CoA dehydratase* (*HACD*) and nine *fatty acid elongase* (*ELO*) genes in the fatty acid elongation pathway; five *fatty acyl-CoA reductase* (*FAR*) genes in the cutin, suberine and wax biosynthesis pathway; and three phospholipase genes in the glycerophospholipid metabolism pathway. RNA-seq and qRT-PCR results confirmed that the knockdown of *NlML1* significantly affected the expression of genes associated with lipid metabolism pathways. In addition, it was confirmed that the transcript levels of the *CYP306A2* gene were significantly downregulated by *NlML1* RNAi in *N. lugens* (figure 7*c*). Subsequently, the tissue-specificity of these genes was investigated. The majority of the genes were highly expressed in integument and/or fat body, while seven genes displayed different expression patterns. The *TGL* (*Nlug19897*) gene was exclusively expressed in the digestive tract; *TGL* (*Nlug22688*), *Elo1*, *Elo2* and *Elo18* were specifically expressed in the testis; while *Elo7* was only expressed in the ovaries (figure 7*d*; electronic supplementary material, figure S5), suggesting that most of the genes are associated with lipid metabolism in the integument and/or fat body.

**Figure 8.**
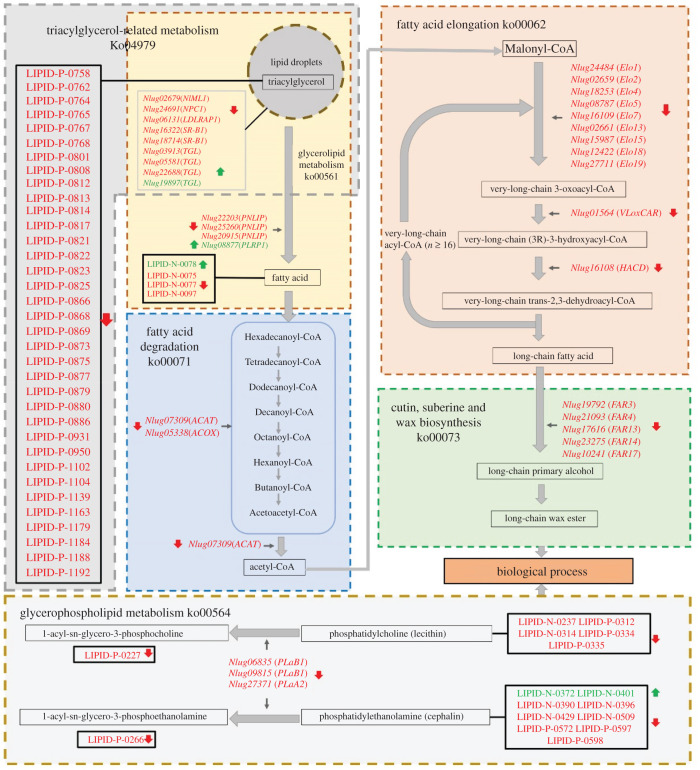
Diagrams depicting the *NlML1*-regulated lipid metabolism pathways. The pathways are shown within the dotted line boxes. Red and green arrows indicate the downregulated and upregulated genes and lipid species, respectively.

Once the validated gene expression profiling was combined with the metabolome datasets, the correlations between DEGs and DLMs were mapped (figure 8). In the TG-related metabolism pathway, seven functional genes—*NPC1*, *LDLRAP1*, two *SR-B1* and three *TGL* genes—displayed significantly downregulated transcript levels, and 36 lipid species belonging to TG presented decreased levels in the ds*NlML1*-injected samples compared with the controls, suggesting that lipid contents are correlated with the transcript abundance of those genes that are required for these pathways. In the glycerolipid metabolism pathway, three *PNLIP* genes and one *PLRP1* gene, which are necessary for fatty acid release from lipids (i.e. TGs) showed significantly down- and upregulated transcript levels, which are associated with the modification of transcripts in four lipid species. In the glycerophospholipid metabolism pathways, one *phospholipase A2* (*PLaA2*) and two *phospholipase B1* (*PLaB1*) genes, which are required for the synthesis of 1-acyl-sn-glycero-3-phosphocholine and 1-acyl-sn-glycero-3-phosphoethanolamine, showed significantly downregulated levels, which are associated with the transcript modification of 16 lipid species. In the fatty acid degradation pathway, acetyl-CoA is known to be the fatty acid metabolite initiating the long-chain acyl-CoA synthesis during fatty acid elongation. Thirteen genes—one *ACAT*, one *ACOX*, nine *Elo*, one *VLoxCAR* and one *HACD—*involved in these two pathways displayed significantly downregulated levels, establishing a correlation between acetyl-CoA and long-chain acyl-CoA synthesis. Furthermore, in the cutin/suberine/wax biosynthesis pathway, five *FAR* genes required for long-chain primary alcohol synthesis had significantly downregulated levels, implying a correlation with long-chain wax ester production. The map obtained from the joint analysis of the transcriptome and metabolome profiling illustrates that *NlML1* knockdown changed the transcript abundance of the multi-genes involved in lipid metabolism pathways and affected the process of lipid accumulation in *N. lugens*.

### 2.8. Analysis of lipid droplets in *Nilaparvata lugens* fat body

**Figure 9.**
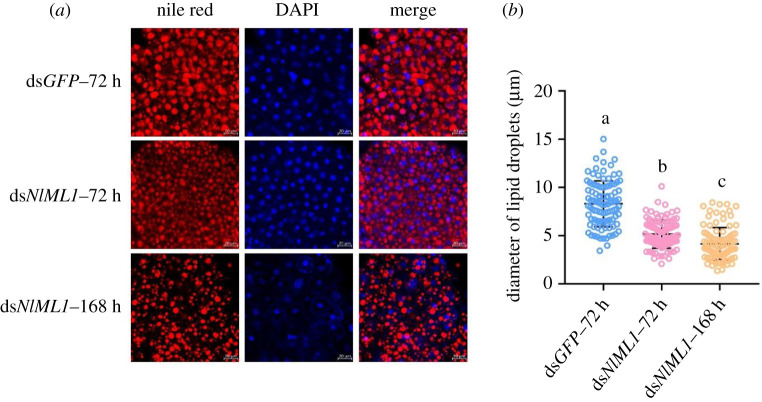
Determination of lipid accumulation in the fat body. (*a*) Morphological observations of lipid droplets in the fat body after *NlML1* knockdown. The 3rd instar nymphs were injected with ds*NlML1* and the fat bodies were isolated from the 5th instar nymphs at 72 h and 168 h after RNAi. Intracellular lipid droplets and nuclei were stained by Nile red and DAPI, shown in the images as red and blue fluorescence, respectively. The ds*GFP*-injected 5th instar nymphs at 72 h were used as negative controls. Scale bar, 20 µm. (*b*) Lipid droplet sizes in the fat body. The diameter of lipid droplets was measured using ImageJ v. 1.52a software (National Institutes of Health, Maryland, USA). The statistical analysis was performed using a one-way ANOVA followed by a Games–Howell *post hoc* test (*p* < 0.05) with SPSS v. 18.0 (mean ± s.d.; *n* = 100). The data were plotted with GraphPad Prism v. 8.

Based on the joint analysis results, the levels of 36 lipid species that belonged to the category of TGs were reduced in the TG-related metabolism pathway. KEGG analysis showed that these TGs were concentrated in lipid droplets. The functional genes known as *TGL*, *PNLIP* and *PLRP1*, which are involved in TG metabolism, had significantly downregulated trasncript levels upon *NlML1* RNAi. To understand if the knockdown of *NlML1* affected lipid accumulation in droplets, the morphological changes and distribution of lipid droplets in the fat bodies of ds*NlML1*-injected 5th instar nymphs were monitored at 72 h and 168 h after RNAi, using Nile red staining. Knockdown of the *NlML1* gene generated lipid droplets that were visibly smaller in size in the fat body cells of the 5th instar nymphs, at both 72 h and 168 h, than those observed in the ds*GFP*-injected controls at 72 h (figure 9*a*). Lipid droplet size in the fat body cells of ds*NlML1*-injected nymphs was 5.2 µm and 4.1 µm at 72 h and 168 h, respectively, while it measured 8.3 µm in the ds*GFP*-injected controls (figure 9*b*). These results suggest that the depletion of the *NlML1* gene affects lipid accumulation in droplets located in the fat body cells.

### 2.9. Effect of *NlML1* RNAi on 20E levels and rescue experiments with 20E injections

**Figure 10.**
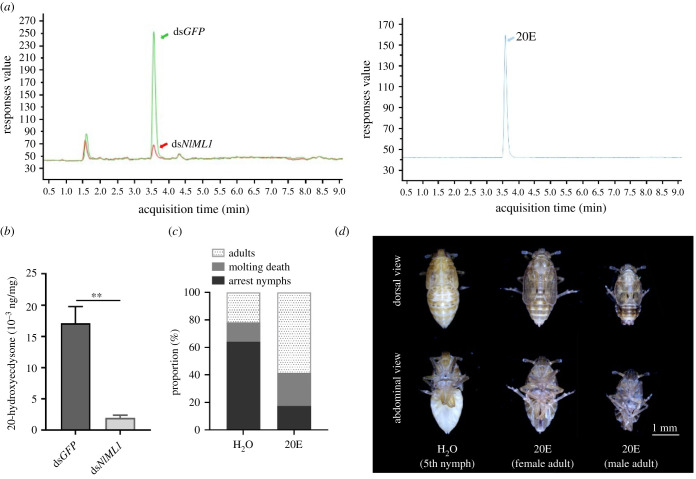
Effects of *NlML1* RNAi on 20E synthesis. (*a*) Detection of 20E in the whole body of 5th instar nymphs. The hormone was extracted from the whole bodies of 5th instar nymphs at 48 h and was determined by LC–MS/MS (left panel). Green and red arrows indicate ds*NlML1*- or ds*GFP*-injected samples, respectively. Chromatograms of the standard 20E solution are shown in the right panel. (*b*) Quantification of 20E in *N. lugens*. The 20E content was quantified in ds*NlML1-* and ds*GFP-*injected 5th instar nymphs at 48 h. The 20E amounts in each dsRNA treatment are represented as the mean per milligram of whole body in three independent experiments. For each experiment, 50 mg of nymph sample was used to quantify the 20E amounts. (*c*) Calculation of the moulting rates of dsRNA-injected 5th instar nymphs after 20E rescue. The 3rd instar nymphs were injected with ds*NlML1* and approximately 0.6 µg of 20E was injected into the ds*NlML1*-treated 5th instar nymphs at 48 h. Moulting and mortality rates were calculated on the 4th day after 20E injection; ddH_2_O with 5% ethyl alcohol was injected as the control. (*d*) Phenotypic observations of dsRNA-injected insects after 20E rescue. The ds*NlML1*-injected nymphs were rescued with ddH_2_O and 20E, respectively. The left panel shows the nymphs injected with ddH_2_O, which were not able to develop into adults and remained at the 5th instar nymph stage; the middle and right panels show the nymphs injected with 20E, which successfully developed into female and male adults.

It was observed that *NlML1* knockdown resulted in the developmental arrest and moulting deficit of nymphs at the 5th instar stage. In addition, qRT-PCR analysis confirmed that the expression of the *CYP306A2* gene was significantly decreased by *NlML1* RNAi. CYP306A2 is a rate-limiting enzyme converting 5β-ketodiol (25,22,2-trideoxyecdysone) to β-ketotriol (22,2-dideoxyecdysone) in the 20E synthesis pathway, and 20E is a key hormone for insect moulting and metamorphosis. Based on these experimental data it was possible to determine whether the decrease of *NlML1* transcription affected the levels of 20E. The highest 20E level had been previously quantified at 48 h in the 5th instar *N. lugens* nymphs [29] and, in the present study, the hormone levels were determined at 48 h in the ds*NlML1*- and ds*GFP*-injected 5th instar nymphs. For the latter, a typical chromatogram of 20E was produced using LC–MS/MS (figure 10*a*, left panel), in the same way as the chromatograms of the standard 20E solution were produced (figure 10*a*, right panel). The MS/MS spectra of the 20E extracted from ds*NlML1*-injected nymphs produced a very small 20E peak (figure 10*a*, left panel), suggesting that ds*NlML1* RNAi significantly reduced the 20E levels of the 5th instar nymphs at 48 h. These levels were very low in the ds*NlML1*-injected 5th instar nymphs at 48 h (0.002 ng mg^−1^) compared with those observed in the ds*GFP*-injected controls (0.017 ng mg^−1^) (figure 10*b*), indicating that the knockdown of *NlML1* significantly affects 20E production. To confirm whether a deficiency in 20E production caused developmental arrest and moulting failure in the 5th instar nymphs, rescue experiments were conducted by injecting 600 ng of 20E in each nymph at 5 dpi following ds*NlML1* RNAi in the 3rd instar nymphs. The phenotypes were observed 4 days after the 20E injection: 64% of the ds*NlML1*-injected nymphs subjected to ddH_2_O treatment underwent developmental arrest at the 5th instar stage for at least 5 days; only 21.7% of the nymphs developed into adults and 14.3% of the nymphs died at the nymph–adult transition (figure 10*c*). By contrast, 58.7% of the ds*NlML1*-injected nymphs subjected to 20E treatment successfully moulted from their split dorsal ecdysial line and developed into adults; 17.3% of the nymphs underwent developmental arrest at the 5th instar stage for at least 5 days, and another 24% were not able to moult and died (figure 10*c*). Morphological observations showed that the ds*NlML1*-injected 5th instar nymphs under ddH_2_O treatment underwent developmental arrest at 10 dpi following ds*NlML1* injection (figure 10*d*, left panel), while the nymphs under 20E treatment completed the nymph–adult transition and developed into female or male adults (figure 10*d*, middle and right panels). These results clearly indicate that 20E rescued the deficient moulting phenotypes derived from ds*NlML1* RNAi.

### 2.10. Effects of *NlML1* knockdown on the genetic expression of integument proteins and on integument formation

**Figure 11.**
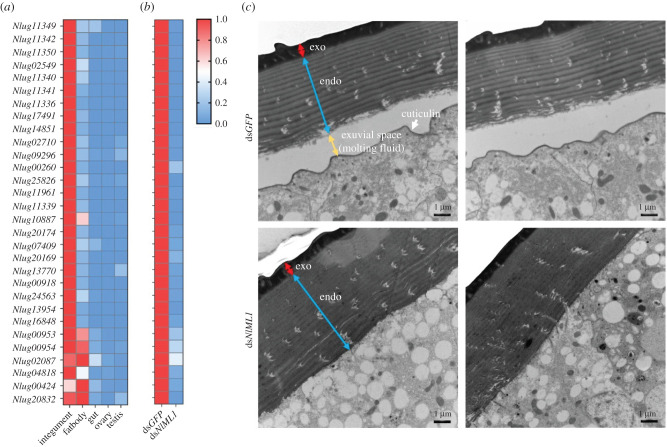
Effects of *NlML1* RNAi on the genetic expression of integument proteins and integument formation. (*a*) Tissue-specific expression of integument protein genes. A heatmap was plotted using GraphPad Prism v. 8 software based on the qRT-PCR data derived from three biological replicates. Total RNAs were extracted from the fat body, gut and integument of the 5th instar nymphs, from the ovaries of female adults and testes of male adults (*n* = 50–100). Relative transcript levels of the target genes in each tissue were determined by qRT-PCR as described in figure 1. The relative transcript levels of each gene in each sample were normalized using *N. lugens β*-*actin* threshold cycle (*C*_t_) values obtained from reactions run on the same plate. Red and blue colours refer to the high and low transcript levels of target gene expression, respectively. The *y*-axis shows the gene ID in the transcriptome and the *x*-axis shows the types of tissue. (*b*) Verification of transcript level variations in integument protein genes. The 3rd instar nymphs were injected with ds*NlML1* or ds*GFP* and total RNAs were extracted from the 5th instar nymphs at 72 h (*n* = 20–30). The relative transcript levels of target genes in each RNAi treatment were determined by qRT-PCR as described above. The relative transcript levels of each gene in each sample were normalized using *N. lugens 18S rRNA* threshold cycle (*C*_t_) values obtained from reactions run on the same plate. A heatmap was plotted using GraphPad Prism v. 8 software based on the qRT-PCR data derived from three biological replicates. Red and blue colours refer to the high and low transcript levels of target gene expression, respectively. The *y*-axis shows the genes ID and the *x*-axis shows the specific dsRNA treatment. (*c*) TEM observations of the integument of dsRNA-injected nymphs. The 3rd instar nymphs were injected with ds*NlML1* or ds*GFP*. The integument was removed from 5th instar nymphs at 72 h. The prefixes ‘endo’ and ‘exo’ refer to endocuticle and exocuticle, respectively. The white arrow indicates the cuticulin. The exuvial space is shown between the endocuticle and cuticulin. Scale bars, 1 µm.

Based on transcriptome annotations, more than 100 DEGs were shown to have a connection with integument proteins. Thirty genes were selected for the analysis of transcript variation in 5th instar nymphs at 72 h (electronic supplementary material, table S8). The tissue expression specificity of these genes was determined by qRT-PCR. Results showed that 24 genes had very high expression levels in the integument, but extremely low levels in the fat body, gut, ovaries and testes; and six genes presented a particularly high expression in the integument and fat body (figure 11*a*; electronic supplementary material, figure S6). In addition, the transcript levels of all these genes were significantly downregulated after the ds*NlML1* injection in 5th instar nymphs at 72 h, compared with the ds*GFP*-injected controls (figure 11*b*; electronic supplementary material, figure S7). In order to investigate the causes of moulting deficits in the 5th instar nymphs, their integument structures were observed at 72 h, which corresponded to the end of the nymphal stage within the 24 h-nymph–adult transition. Transmission electron microscope (TEM) observations showed that ds*GFP*-injected nymphs presented normal integumental structures, which consist of exocuticle, endocuticle, moulting fluid and cuticulin (in order, moving from the outside toward the inside) (figure 11*c*). Generally, when nymphs are undergoing the nymph–adult transition, moulting fluids are secreted into the apolysial space by the epidermal cells between the old endocuticle and the cuticulin, leading to the formation of the new integument [30]. The moulting fluid was observed between the old endocuticle and cuticulin in the ds*GFP*-injected nymphs. However, ds*NlML1* knockdown caused the absence of both moulting fluid and cuticulin in the integument, possibly impeding the formation of new integument.

### 2.11. Effect of 20E rescue on the expression of DEGs in ds*NlML1*-injected nymphs

**Figure 12.**
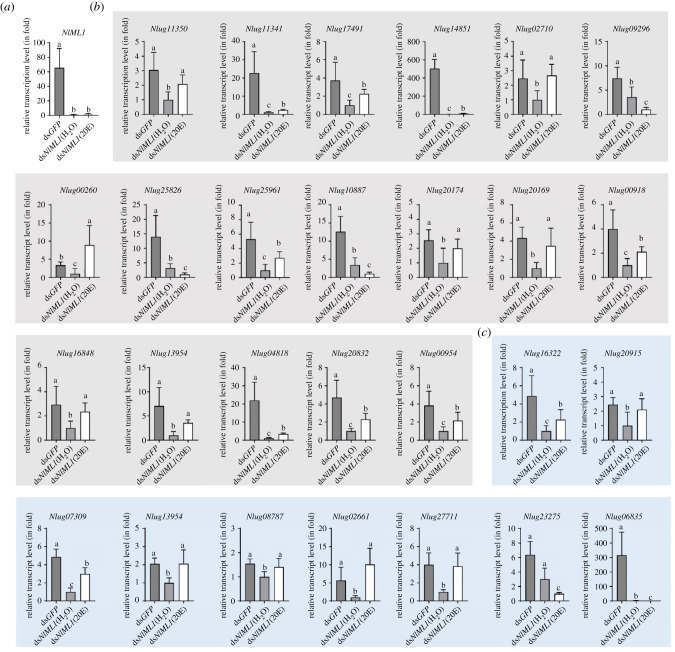
Investigation of the transcript level variations of DEGs by 20E treatment in ds*NlML1*-injected nymphs. (*a*) Determination of the transcript level variations of *NlML1* gene after 20E injection. The 3rd instar nymphs were injected with ds*NlML1* or ds*GFP* and approximately 0.6 µg of 20E was injected into the ds*NlML1*-injected 5th instar nymphs at 48 h. Total RNAs were extracted from *N. lugens* nymphs on the 2nd day after 20E injection and the transcript levels of each DEGs were analysed by qRT-PCR, as described in figure 1. The relative transcript levels of each gene in each sample were normalized using *N. lugens 18S rRNA* threshold cycle (*C*_t_) values obtained from reactions run on the same plate. (*b*) Verification of the transcript level variations of the integument protein genes after 20E injection. (*c*) Verification of the transcript level variations of the lipid and chitin metabolism enzyme genes. The statistical analysis was performed using a one-way ANOVA followed by a Tukey's or Games–Howell *post hoc* test (*p* < 0.05).

As 20E treatment rescued the moulting deficits derived from ds*NlML1* RNAi, we investigated whether 20E affects the transcript levels of DEGs in ds*NlML1*-injected nymphs. We selected 63 DEGs, which were confirmed to have significantly differential expressions as shown in figure 7*c* and figure 11*b*, to measure their transcript level variation after 20E injection. We found that 20E injection did not alter *NlML1* transcript levels in ds*NlML1*-injected 5th instar nymphs when compared to the ddH_2_O-injected controls (figure 12*a*). However, 20E but not ddH_2_O significantly enhanced the transcript levels of 22 DEGs in ds*NlML1*-injected nymphs (figure 12*b*,*c*). They include 15 integument protein and 7 lipid and chitin metabolism enzyme genes. Among these genes, two chitinases (*Nlug04818* and *Nlug20832*) and one chitin deacetylases (*Nlug00954*) are expected to be the components of the moulting fluid in the nymphs. The investigations suggest that 20E regulates the gene expressions of a partial of integument proteins, lipid and chitin metebolism enzymes in the 5th instar nymphs. Their significantly increased transcript levels by 20E may explain the observations that 20E rescued deficient moulting phenotypes. In addition, 20E significantly reduced the transcript levels of 5 integument-specific genes (*Nlug09296*, *Nlug25826*, *Nlug10887*, *Nlug23275* and *Nlug06835*) (figure 12*b*,*c*); while did not alter the transcript levels of 36 DEGs in ds*NlML1*-injected nymphs, implicating that the expression of these genes were not regulted by 20E. These results indicate that *NlML1* is an upstream gene in the 20E biosynthestic pathway and regulates 20E synthesis. On the other hand, 20E regulates the gene expression of some integument proteins and lipid and chitin metebolism enzymes that are important for moulting and emergence of the *N. lugens* nymphs.

## 3. Discussion

In this study, three ML domain-containing genes with similar nucleotide length were identified in *N. lugens*. The three-dimensional model structures showed that they all have two antiparallel β-sheets and a hydrophobic cavity, probably used for binding the lipids. Despite having a characteristic ML domain, the percentage of amino acid sequences they share is low, between 11% and 20%. The NlML1 and NlML2 proteins contain a predicted N-terminal signal peptide, indicating the presence of secretory proteins. The protein sequences include eight and six conserved cysteine residues, respectively, which may be involved in the formation of intermolecular disulfide bridges. Based on the predicted structures of the NlML1 and NlML2 proteins, the nearest bridge distances connecting each two cysteines in the NlML1 or NlML2 protein were greater than 2.4 Å, which is longer than the 2.1 Å required to form a disulfide bridge, and intramolecular disulfide bonds therefore could not be predicted. NlML3 lacks a signal peptide but it contains seven cysteine residues; it is believed that these form three intramolecular disulfide bridges with the 2.0 Å or 2.1 Å required to form the disulfide bridges connecting each two cysteines, which is consistent with the formation of three disulfide bonds observed in the MD-2 proteins in humans and mice [12,31]. Phylogenetic tree analysis indicates that MLs in *N. lugens* present diverged evolutionary relationships. NlML1 is closely related to insect MD-2 proteins that have eight conserved cysteine residues, but is distant from the MD-2 proteins of human and mice, which are characterized by six conserved cysteines. NlML2 forms an independent branch with a few of homologues that are limited in *B. mori* and *D. melanogaster*. NlML3 forms another independent branch, showing close relationships with the NPC2 proteins of human and mice, while being distant from their MD-2 proteins. Temporospatial analysis further indicates that *ML* genes in *N. lugens* have different expression specificities; for example, *NlML1* is highly expressed in fat bodies. This observation is consistent with the expression patterns of the *ML* homologues observed in mammalian, shrimp and insect species. Unexpectedly, *NlML1* transcripts were detected at high levels in laid eggs, suggesting they may have a functional role in the reproductive process. By contrast, *NlML2* and *NlML3* were lowly expressed in the fat bodies and eggs but highly expressed in testis or gut, indicating that these two genes differ from *NlML1* in terms of physiological functions. To understand the biological roles of *NlML*s, RNAi was performed by knocking down their transcript levels in *N. lugens*. The knockdown of *NlML2* and *NlML3* did not generate abnormal developmental or reproductive phenotypes and produced a satisfactory RNAi effect. However, *NlML1* knockdown led to the apparent developmental arrest at the 5th instar nymph stage and lethal deficits in the moulting transition from nymph to adult. The nymphs that underwent arrest remained at the 5th instar stage for at least 5 days, and in one record case for 31 days. A comparison with the control individuals revealed that the arrested female nymphs had non-fully developed, small-sized ovaries. Interestingly, these female nymphs displayed a fully developed ovipositor under the abdomen integuments, while the control individuals did not. Morphological observations showed that the ds*NlML1*-injected individuals had the characteristics of both the 5th instar nymph and female adult, suggesting that the nymphs underwent dysecdysis at the moulting transition from nymph to adult. The location of NlML1 proteins was identified in the fat bodies, ovaries and laid eggs. Immunofluorescence staining clearly detected specific NlML1 signals, mainly in the membranes of the fat body cells and of the follicular epithelial cells found in the ovaries; in the laid eggs, NlML1 proteins were located outside the nuclei of the blastoderm cells. These observations suggest the possibility that NlML1 mediates lipid recognition in the fat bodies, and may, therefore, play an important role in oocyte maturation, egg laying and hatching. A further western blotting assay indicated that NlML1 is present in two forms: a secreted monomeric protein and an oligomeric protein on the cell surface. A single specific protein band of about 17 kDa was detected in the haemolymph of female adults, and its size is consistent with the theoretical molecular mass of NlML1. Protein bands of approximately 34 kDa were visualized in the fat body, ovary, laid egg, integument and whole-body samples of both nymphs and adults. The detection of proteins with a size of 34 kDa, which is double the 17 kDa size, suggests that NlML1 may form a dimer via interchain disulfide bonds on the cell membranes of fat body, integument and follicular epithelial cells and in eggs; a process which may mediate lipid signal transduction pathways. As the NlML1 protein has a predicted signal peptide, it is suggested that the 17 kDa protein is a free monomer secreted into extracellular spaces such as the haemolymph. As the NlML1 protein was detected in the ovaries and eggs, its physiological function in female adult reproduction was investigated. It was observed that RNAi blocked the laying of mature oocytes on rice plants, also significantly decreasing hatching rates. To explain this observation, RNA-seq and lipid metabolomic profiling analyses were performed by comparing *NlML1*-knockdown specimens with controls. GO and KEGG analyses revealed that *NlML1* was associated with cuticle development and chitin-based cuticle structure. In particular, *NlML1* regulated the expression of genes that are enriched in lipid metabolism signalling pathways, including fatty acid degradation, elongation, production of unsaturated fatty acids, fat digestion and absorption, linoleic acid metabolism and glycosphingolipid biosynthesis pathways. A joint analysis of transcriptome and lipid metabolome further verified the correlation between the differently expressed genes and lipid species. Based on 27 overlapping pathways between the transcriptome and metabolome profiling, a *NlML1*-regulated gene network was established, once the differential gene expression was confirmed. In this network, the silencing of *NlML1* significantly downregulated the expression of genes involved in TG-related metabolism, glycerolipid and glycerophospholipid metabolism, fatty acid degradation/elongation and cutin/suberine/wax biosynthesis. These results provide omics evidence indicating that *NlML1* is associated with lipid metabolism regulation.

Through qRT-PCR analysis, it was revealed that the *NlML1* gene was highly expressed in the fat body, which plays an essential role in energy storage and utilization in insects. Lipids are the primary component of the fat body, representing more than 50% of the dry weight [24]. A great amount of lipid reserves, mostly triglycerides, are stored as cytoplasmic lipid droplets in fat body cells and serve as resources to sustain energy demands for insect growth and reproduction [25]. Experimental evidence demonstrated that *NlML1* knockdown generates smaller lipid droplets in the fat body cells. This fact and the decreased levels of 36 lipid species of triglycerides observed in the metabolome profiling both suggest that *NlML1* is involved in lipid metabolism processes occurring in the fat body. *NlML1* knockdown significantly downregulated the expression of the genes encoding NPC1, LDLRAP1, two SR-B1 proteins and three TG lipases, and significantly upregulated the expression of a *TGL* gene involved in TG-related metabolism pathways. NPC proteins are essential for sterol homeostasis in eukaryotic cells. In humans, disturbances of NPC signalling pathways lead to Niemann–Pick disease type C, a neurodegenerative disease where cholesterol and other lipids accumulate in the lysosomes [32]. *Drosophila* has eight Npc2 proteins (Npc2a–h), of which Npc2a has the highest sequence similarity to human NPC2. Single *npc2a* or *npc2b* mutants were viable and fertile, with no developmental delay. However, most *npc2a*; *npc2b* double mutants were not viable, but could be rescued by feeding with a diet enriched in cholesterol, 7-dehydrocholesterol or 20E [33]. In mammals, SR-B1 is a cell-surface receptor binding to both high- and low-density lipoproteins. In addition to binding to lipoproteins, this receptor can mediate the uptake of lipids other than cholesterol, including phospholipids and triglycerides [34,35]. There is currently very little information about the expression, structural organization and function of SR-B1 in insects [36]. However, the SR-B1 family was shown to be involved in steroidogenesis by regulating cholesterol uptake in the prothoracic gland, required for the synthesis of ecdysone in *Drosophila* [37], and SR-B1 played important roles in the innate immune response in *Plutella xylostella* [38]. Unexpectedly, in the present study it was observed that most of these verified DEGs had very high transcript levels in the integument and/or fat body, but extremely low levels in the other tested tissues, indicating that the *NlML1* gene regulates lipid metabolism, transportation or accumulation in the fat body and integument.

The hormone known as 20E is an active steroid that regulates many aspects of the developmental process and reproductive activity in insects. Its synthesis is initiated by cholesterol and is regulated by a series of ecdysteroidogenic enzymes. The *CYP306A2* gene, encoding the key rate-limiting enzyme in the 20E biosynthesis pathway, showed significantly decreased transcript levels in ds*NlML1*-injected nymphs. Our results clearly demonstrated that *NlML1* knockdown significantly reduced 20E levels in the 5th instar nymphs at 48 h, while 20E injections counteracted the moulting deficits derived from the depletion of *NlML1*. This hormone also regulates oocyte development and germ stem cell maintenance in insects [39–41]. In our previous study, we demonstrated that 20E is required for egg production and hatching in *N. lugens* [29] and, in this study, it was observed that *NlML1* knockdown inhibited egg laying and hatching. This finding establishes possible links between *NlML1* expression, 20E production and egg development in *N. lugens* females. Further studies are required to investigate embryonic ecdysteroidogenesis in hemipteran insects, to improve our understanding of the universal system of embryonic ecdysteroidogenesis among insects.

*NlML1* knockdown significantly downregulated gene expression in fatty acid metabolism pathways, in particular the expression of three genes encoding PNLIP, which are required for fatty acid release from lipids; two genes encoding ACAT and ACOX, required for the production of acetyl-CoA in the fatty acid degradation pathway; nine genes encoding ELO and two genes encoding VLoxCAR and very-long-chain HACD, which catalyse the synthesis of long-chain acyl-CoA in the fatty acid elongation pathway; and finally, five genes encoding alcohol-forming FAR, required for the production of long-chain wax ester in the cutin, suberine and wax biosynthesis pathways. qRT-PCR revealed that more than half of the genes related to fatty acid metabolism were highly expressed in the integument of *N. lugens*, indicating that *NlML1* is associated with integument function. The ELO and FAR enzymes are essential for catalysing the synthesis of long-chain fatty acids and fatty alcohols that are precursors of sex pheromones, wax ester and cuticular hydrocarbons. In *N. lugens*, *ELO* and *FAR* genes play crucial roles in moulting, integument waterproofing and adult fertility in females [42,43]. In this study, the moulting deficits generated by *NlML1* knockdown were the same as those observed in *FAR* or *ELO* deficient *N. lugens* individuals. These findings suggest that *NlML1* is involved in integument functions through the regulation of the synthesis and metabolism of long-chain lipids.

In addition to the genes related to fatty acid metabolism, this study identified three *NlML1*-downregulated genes encoding one PLaA2 and two PLaB1, which are key enzymes for the synthesis of 1-acyl-sn-glycero-3-PC and 1-acyl-sn-glycero-3-PE in the glycerophospholipid metabolism pathway. The decreased transcript levels of *PLaA2* and *PLaB1* are tightly coupled with the reduced levels of 14 lipid species of PC, PE, 1-acyl-sn-glycero-3-phosphocholine and 1-acyl-sn-glycero-3-phosphoethanolamine. Tissue expression analysis showed that the *PLaA2* and *PLaB1* genes were exclusively expressed in the integument. These findings suggest that 1-acyl-sn-glycero-3-PC and 1-acyl-sn-glycero-3-PE, which are required for moulting, could be produced from PC and PE in the integument.

The integument structures of the ds*NlML1*-injected 5th instar nymphs were examined at 72 h, which corresponds to the end of the nymphal stage before the transition into adult. An accumulation of moulting fluids was observed in the exuvial space in the ds*GFP*-injected control individuals. In *N. lugens*, moulting fluid is synthesized and secreted in the exuvial space by epidermal cells between the old endocuticle and cuticulin, and in this space, the formation of new integument will take place during the moulting process [44]. However, in ds*NlML1*-injected nymphs, the exuvial space was not formed due to the failure in both the accumulation of moulting fluids and cuticulin formation. In *B. mori*, the enzymes related to chitin metabolism and involved in the degradation of integument proteins and chitin, are rich in moulting fluids before pupation and eclosion [45]. Our study revealed that gene expression of the chitin metabolism-related proteins, including three chitin deacetylases and three chitinases, were significantly downregulated by *NlML1* knockdown in *N. lugens*. These genes were found to be highly expressed in the integument and they are expected to regulate the synthesis of moulting fluids because their homologous enzymes were found to be abundant in the moulting fluids of *B. mori*. Moreover, the expression of more than one hundred genes associated with the synthesis of integument proteins in insects was shown to be significantly downregulated by *NlML1* knockdown. A number of these proteins correspond to the homologous proteins identified in the moulting fluids of *B. mori*. In *N. lugens*, the considerable inhibition of gene expression in the enzymes related to chitin metabolism and in integument proteins could be associated with the absence of moulting fluids and cuticulin. This may explain the failure in both the formation of new integument and degradation of the old one, which led to moulting failure.

In conclusion, the *NlML1* gene plays very important roles in regulating lipid metabolism in *N. lugens*. Our findings revealed that *NlML1* is required for TG-related metabolism and it mediates 20E synthesis. In addition, it is also necessary for long-chain lipid synthesis and glycerophospholipid metabolism. Most of the downregulated genes involved in these metabolic pathways presented very high transcript levels in the integument. It is concluded that *NlML1* contributes to the formation of new integument and to the degradation of the old one, which are two key phases ensuring moulting success. This gene also plays an important role in ensuring the successful hatching of laid eggs. Our study of the *NlML1*-regulated lipid metabolic network provides valuable information for an improved understanding of the mechanisms of moulting, metamorphosis, development and reproduction in *N. lugens*. The results of this study will assist the production of potential target genes to be used for the future management of rice pests, and they will also contribute to recognizing the importance of ML proteins for human health.

## 4. Material and methods

### 4.1. Insects

*Nilaparvata lugens* individuals were originally collected from a rice field in the Huajiachi Campus of Zhejiang University, Hangzhou, China in 2008 and were subsequently maintained in the laboratory on a diet consisting of fresh rice seedlings (*Oryza sativa* strain Xiushui 110) at 26 ± 0.5°C and 50 ± 5% humidity, under a 16 l : 8 d h photoperiod, as described previously [23].

### 4.2. Bioinformatics analysis

*NlML* sequences were searched against the *N. lugens* genome (GenBank accession number GCA_014356525.1, http://insect-genome.com/planthoppers/) and the transcriptome (accession number SRX023419) was obtained from the Sequence Read Archive database (http://www.ncbi.nlm.nih.gov/sra) using the National Center for Biotechnology Information (NCBI) reference sequences (https://www.ncbi.nlm.nih.gov/refseq/). The amino acid sequences were predicted by DNASTAR Lasergene EditSeq (https://www.dnastar.com/software/lasergene/). Signal peptides were predicted by SMART (http://smart.embl.de/) and *N*-glycosylation sites were predicted by DTU Health Tech (https://services.healthtech.dtu.dk/). The amino acid sequence alignments were performed using ClustalX (http://www.clustal.org/). The deduced domain structures were determined using SMART and Pfam (http://pfam.xfam.org/). A phylogenetic tree was constructed using the maximum-likelihood method in Mega X (http://www.megasoftware.net/). Phylogenetic relationships were determined using bootstrap analysis values derived from 1000 trials.

### 4.3. Quantitative real-time PCR (qRT-PCR)

Total RNAs were extracted from each developmental stage and each tissue using an RNAiso plus Kit (TaKaRa) and concentrations were determined using a NanoDrop 2000 Spectrophotometer (Thermo Fisher Scientific). First-strand cDNA was synthesized using a Hiscript II Q RT SuperMix for qPCR (+gDNA wiper) Kit (Vazyme) to remove any contaminating genomic DNA residues. A no-template control (RNA with no-reverse-transcriptase) was used to detect contamination. Subsequently, qRT-PCR reactions were run on a CFX Connect Real-Time System (Bio-Rad, Hercules, CA, USA) using ChamQ SYBR qPCR Master Mix Kit (Vazyme) under the following conditions: denaturation for 3 min at 95°C followed by 40 cycles of two-step PCR for 15 s at 95°C and 30 s at 55°C. One microgram of starting RNA was used for reverse transcription in a 20 µl reaction and 2 µl of the first-strand cDNA (diluted 10 times) was analysed in each 20 µl reaction by qRT-PCR. The gene-specific primers were designed using the Primer Premier 6.0 program based on the *N. lugens* transcriptomic sequences, as shown in electronic supplementary material, table S9. The *N. lugens* house-keeping genes known as *18S ribosomal RNA* (*18S rRNA*) and *β*-*actin* (GenBank accession no. JN662398 and XP_022202043) were used as the internal controls. The results (threshold cycle values) of the qRT-PCR assays were normalized to the transcript levels of internal genes that were obtained from reactions run on the same plate. The 2^−ΔΔCt^ method (Ct represents the cycle threshold) was used to measure relative transcript levels [46]. In each assay, the transcript level was normalized to the lowest level, which was arbitrarily set at one. Three biological replicates were performed and, for each one, three technical replicates were conducted.

### 4.4. Sample collection for developmental stage- and tissue-specific expression analyses

To investigate the expression levels of *NlML* genes at different developmental stages, samples were collected from mixed eggs at different time points after laying (1 h, *n* = 20; 24 h, *n* = 20; 48 h, *n* = 20; 72 h, *n* = 20; 96 h, *n* = 20; 120 h, *n* = 20; 144 h, *n* = 20; 168 h, *n* = 20), first-instar (*n* = 100), second-instar (*n* = 50), third-instar (*n* = 50), fourth-instar (*n* = 30) and fifth-instar nymphs (*n* = 20), and male (*n* = 20) and female adults (*n* = 20). Each developmental stage sample was individually pooled as one sample. For tissue-specific analysis, fat body (*n* = 50), ovary (*n* = 30), integument (*n* = 50), salivary gland (*n* = 100) and gut (*n* = 50) were dissected from female adults, and testis (*n* = 50) was dissected from male adults using an S8AP0 stereomicroscope (Leica Microsystems GmbH, Wetzlar, Germany), and were washed in a diethylpyrocarbonate-treated NaCl/Pi solution (pH 7.4), as described previously [28]. Each tissue sample was pooled as one sample. Three biological replicates were conducted for each sample collection.

### 4.5. Double-stranded RNA synthesis and RNAi

Three *NlML* genes were individually amplified and cloned into the pMD-19T vector (TaKaRa, Dalian, China). Double-stranded RNAs (dsRNA) were synthesized *in vitro* through PCR-generated DNA templates using a T7 high yield RNA Transcription Kit (Vazyme, Nanjing, China). A 493 bp coding sequence of a green fluorescent protein (GFP) derived from *Aequorea victoria* was used as the control dsRNA (ds*GFP*). The dsRNAs were quantified using a NanoDrop 2000 Spectrophotometer (Thermo Fisher Scientific, Bremen, Germany). The specific primers used for the dsRNA synthesis are shown in electronic supplementary material, table S9. After being anaesthetized via carbon dioxide (CO_2_), each third-instar nymph was microinjected into the abdomen with approximately 250 ng of dsRNA using a FemtoJet microinjection system (Eppendorf-Netheler-Hinz, Hamburg, Germany). For reproduction studies, each fifth-instar nymph was microinjected with 300 ng of dsRNA. The injected insects were then reared on fresh rice seedlings at 26 ± 0.5°C and 50 ± 5% humidity under a 16 l : 8 d h photoperiod. Phenotypes were observed following RNAi.

### 4.6. Specific antibody preparation

A 510-bp coding region of the *NlML1* gene was amplified via PCR using a pair of primers flanked with *EcoR* I/*Not* I restriction enzyme sites (electronic supplementary material, table S9). The PCR products and pGEX-6P-1 expression vector were digested with *EcoR* I and *Not* I enzymes for 1 h at 37°C. Subsequently, the coding region of *NlML1* was ligated into the pGEX-6P-1 vector using a DNA Ligation Kit (TaKaRa) overnight at 16°C and transformed into the *Escherichia coli* strain Rosetta (Novagen, San Diego, CA, USA). Protein expression was induced under 1 mM of isopropyl-beta-d-thiogalactopyranoside for 6 h at 37°C. The expressed proteins were separated by SDS–PAGE and the target protein bands were retrieved and dissolved in phosphate-buffered saline (PBS). The polyclonal rabbit antiserum against NlML1 was prepared by the HuaAn Biotechnology Co. (Hangzhou, China).

### 4.7. Immunofluorescence staining

The ovaries and fat bodies were removed from adult females on the 3rd day after emergence. After being washed three times with 1× PBS containing 0.1% Triton X-100 (0.1% PBST), the tissues were fixed in 4% paraformaldehyde for 30 min at room temperature. The eggs were collected 88 h after they were laid in rice leaf sheaths and were treated with 0.5% NaClO buffer (0.6M NaCl, 0.3M NaOH, 10% NaOCl) for 20 min. The eggs were washed three times with 1× wash buffer (0.12M NaCl, 0.05% Triton X-100) and fixed in fixation buffer (37% methanal/n-heptane 1 : 1) by gentle mixing for 5 min and then by more forceful mixing for 20 min. Then, the eggs were transferred to the buffer (methanol/n-heptane 1 : 1) and shaken thoroughly for 15 min. Once the buffer was removed, the eggs were transferred to 100% methanol and shaken again for 15 min. They were then moved to the buffer (methanol/n-heptane 1 : 1) for another shaking phase of 20 min, and were subsequently washed three times in 0.1% PBST for 10 min. Finally, the egg shells were removed using dissecting forceps. The ovaries, fat bodies and the eggs without shells were washed three times in 0.1% PBST, then blocked and permeabilized using 5% normal goat serum in 0.1% PBST for 2 h. A rabbit anti-NlML1 polyclonal antibody was added to detect the NlML1 protein at a dilution of 1 : 250, and the solution was kept overnight at 4°C. After being washed three times in 0.1% PBST, the tissues or the eggs were incubated with Cy3-labelled secondary goat anti-rabbit IgG (Beyotime, Shanghai, China) at a dilution of 1 : 500 for 2 h at room temperature. Subsequently, the samples were stained with 3,3′-dioctadecyloxacarbocyanine perchlorate (DiO, Beyotime, Shanghai, China) for 20 min, and were washed three times in 0.1% PBST. They were then stained with 1 µg ml^−1^ of 4′,6-diamidino-2-phenylindole (DAPI, Thermo Fisher Scientific, Bremen, Germany) for 15 min and washed three times in 0.1% PBST, each time for 10 min. The fluorescent signals were observed under a Zeiss LSM 800 confocal laser microscopy (Carl Zeiss MicroImaging, Göttingen, Germany) and the fluorescence images were analysed with ZEN 2.3 (Carl Zeiss MicroImaging).

### 4.8. Functional analysis of *NlML1* gene during ovulation and egg hatching

Ovulation and egg hatching experiments were conducted according to Wang *et al*. [28]. Briefly, the 5th instar nymphs were microinjected with each ds*NlML1* or ds*GFP*. Brachypterous females have higher fecundity than macropterous females, and we, therefore, used brachypterous female *N. lugens* for ovulation experiments in this study. Newly emerged brachypterous females and males reared on fresh rice seedlings for 1 day. To ensure successful mating, a single dsRNA-injected female was mated with two dsRNA-injected adult males in a long glass tube containing three-leaf stage fresh rice seedlings (9 ± 0.5 cm long) at 26 ± 0.5°C and 50 ± 5% relative humidity under a 16 l : 8 d h photoperiod for 6 days. The adults were then removed and hatched nymphs were observed and counted at 24-h intervals, and were removed from the rice seedlings 11 days later. Unhatched eggs were dissected and counted. The banana-shape oocytes at the bottom of the ooecia were identified as mature oocytes [47]. Biological replicates were carried out for each mating (*n* = 15 females × males).

### 4.9. Identification of *ML* regulated genes via high-throughput RNA-seq

The 3rd instar nymphs were injected with ds*NlML1* or ds*GFP* and total RNAs were extracted from the 5th instar nymphs at 72 h. One microgram of each RNA sample was subjected to RNA-seq analysis. The cDNA library construction and Illumina sequencing were performed at the MetWare Biotechnology laboratories (Wuhan, China). Three biological replicates were carried out for either the ds*NlML1* or ds*GFP* treatment. Clean reads were aligned to the reference genome (GCA_014356525.1) using HISAT2 [48] and the analysis of differential gene expression was performed using the DESeq2 package [49]. Genes with an FDR <0.05 and |log_2_(FC)| > 2 by DESeq2 were categorized as DEGs.

### 4.10. Lipid extraction of *Nilaparvata lugens* whole bodies for lipidomic profiling

The 3rd instar nymphs were injected with ds*NlML1* or ds*GFP* and the total lipids were extracted from the 5th instar nymphs at 72 h as described in [50,51]. In brief, 50 mg of their whole body was homogenized using steel balls in a 1 ml mixture (methyl-*tert*-butyl ether and methanol v/v = 3 : 1), followed by vortexing for 2 min and ultrasonic treatment for 5 min in an ice-water bath. After adding 500 µl of H_2_O, the mixture was vortexed for 1 min and then centrifuged at 12 000*g* for 10 min at 4°C after which 500 µl of the supernatant was collected and evaporated under a stream of N_2_. The lipid extracts were redissolved in a mixture of acetonitrile containing 0.04% acetic acid for LC–MS/MS analysis.

The lipidomic profiling of the sample extracts was conducted at the MetWare Biotechnology laboratories using a liquid chromatography–electrospray ionization–tandem mass spectrometry system (LC–ESI–MS/MS) containing ultra performance liquid chromatography (UPLC) (Shim-pack UFLC Shimadzu CBM30A) and MS/MS (Applied Biosystems 4500 QTRAP). Chromatographic separation was performed on an Acquity UPLC HSS T3 C18 column (1.8 µm, 2.1 mm × 100 mm, Waters, USA) with a flow rate of 0.4 ml min^−1^ and a column temperature of 40°C. Five microliters of sample were injected into the column and eluted with the solvent system consisting of a mobile phase A with ultrapure water (0.04% acetic acid), and a mobile phase B with acetonitrile (0.04% acetic acid). The elution gradient programme was set stepwise as follows: ultrapure water/acetonitrile, 95 : 5 (v/v) 0 min; 5 : 95 (v/v) 11.0 min; 5 : 95 (v/v) 12.0 min; 95 : 5 (v/v) 12.1 min and 95 : 5 (v/v) 14.0 min. The qualitative and quantitative analysis of lipid profiling was performed through multiple reaction monitoring analysis.

### 4.11. Analysis of lipid droplets in *Nilaparvata lugens* fat bodies

Lipid droplets were visualized by staining the fat bodies with Nile red (Sangon Biotech, Shanghai, China) as described previously [52]. In brief, fat bodies were collected from the ds*NlML1*-injected 5th instar nymphs at 72 h and 168 h and washed three times in 1× PBS after which they were fixed in 4% paraformaldehyde for 30 min at room temperature. After being washed three times with PBS containing 0.1% Triton X-100 (PBST), the fat bodies were incubated in 0.1% PBST buffer with 1 µg ml^−1^ Nile red solution for 90 min at room temperature in order to stain the lipid droplets. For nuclear staining, the fat bodies were incubated for 15 min in 1 µg ml^−1^ DAPI at room temperature, and were subsequently washed with 0.1% PBST three times. Finally, the fat bodies were washed twice with 1× PBS and transferred to microslides, and lipid droplets were observed under an LSM 800 confocal microscope (Carl Zeiss MicroImaging). The absorption and emission wavelengths associated with the lipid droplets and nuclei were 550/570 nm and 358/461 nm, respectively. The confocal images were analysed with ZEN v. 2.3 software (Carl Zeiss MicroImaging) and the diameters of the lipid droplets were measured using ImageJ v. 1.52a software (National Institutes of Health, Maryland, USA).

### 4.12. Transmission electron microscopy

The 3rd instar nymphs were injected with ds*NlML1*. The tergum integument of the 5th somite of the abdomen was removed from the 5th instar nymphs at 72 h. The samples were fixed with 2.5% (v/v) glutaraldehyde in PBS (0.1 M, pH 7.0) overnight at 4°C. After being washed three times (for 15 min each time) in PBS, the samples were post-fixed with 1% (v/v) osmium tetroxide (OsO_4_) in PBS for 2 h at room temperature, and were subsequently washed again three times in PBS. The samples were dehydrated through a graded series of ethanol (30, 50, 70, 80, 90 and 95%, v/v) for 15 min each and by exposure to 100% alcohol for 20 min; then they were transferred to acetone for 20 min, embedded in Spurr resin and polymerized for 16 h at 70°C. Semi-thin sections were cut using Leica EM UC7 ultramicrotome (Leica, Germany) and observed using a Hitachi Model H-7650 transmission electron microscope (Hitachi, Tokyo, Japan).

### 4.13. Measurement of 20-hydroxyecdysone and rescue experiments with 20E injections

The whole bodies were used to measure 20E, following protocols described previously [29]. In brief, 50 mg of the 5th instar nymphs at 48 h were homogenized and sonicated for 10 min in 200 µl of sonicated buffer at pH 7.5, containing 50 mM Tris–HCl, 150 mM NaCl and 2 mM EGTA. The 1-butanol (two-fold volume) was added into the samples to extract ecdysteroids by vortexing the solution for 5 min and centrifuging for 10 min at 10 000*g* and 4°C. In order to fully extract 20E from the nymphs, three repeats with the 1-butanol treatment were conducted. The supernatants were collected and evaporated in a centrifugal evaporator (Eppendorf, Hamburg, Germany). For the measurement of 20E in *N. lugens*, steroids were resolved in 100 µl of 100% methanol: ddH_2_O (1 : 1, v/v) and centrifuged for 10 min at 15 000*g* and 4°C. The supernatants were then transferred to a glass insert vial for liquid chromatography–tandem mass spectrometry (LC–MS/MS) as described previously [29]. The presence of steroids in the aliquots was detected using an Agilent 6460 triple quadrupole mass spectrometer (Agilent Technologies, USA) equipped with an ESI source and operated in positive mode. HPLC separation was performed on a Zorbax SB C18 column (3.5 µm, 2.1 ⌀ × 150 mm, Agilent Technologies, CA, USA). The mobile phases consisted of formic acid: water (1 : 1000, v/v; solution A) and acetonitrile (solution B). The gradient programme was set as follows: 10–90% B in 10 min, flow rate at 0.3 ml min^−1^; 20E was detected in MRM mode by monitoring the transitions 481.6 > 445.2.

Subsequently, 250 ng of ds*NlML1* was injected into the 3rd *N. lugens* nymphs (the ds*GFP*-treated nymphs were used as controls) and the 5th instar nymphs at 48 h were injected with 0.6 µg of 20E (Aladdin, Beijing, China). The phenotype was observed approximately 4 days later and 20E was measured from the whole bodies, as described above. The non-treated or ddH_2_O-treated dsRNA-injected nymphs were used as controls in the rescue experiments.

### 4.14. Statistical analysis

Differences between two conditions were analysed by Student's *t*-tests. Multiple comparisons were analysed using one-way ANOVAs followed by the Tukey's or Games–Howell *post hoc* test. The normality of data was tested using the Kolmogorov–Smirnov test (*p* < 0.05), and the equality of variances was tested by Levene's test (*p* < 0.05). Tukey's *post hoc* test was used when normality and variances were homogeneous, and Games–Howell *post hoc* test was used when non-normality and/or variances were heterogeneous. All data analysis was carried out with IBM SPSS Statistics (International Business Machines Corporation). All data are shown as mean ± standard deviation (s.d.). The significantly differential lipids observed between the ds*NlML1-* and ds*GFP*-treated groups were screened out from the orthogonal partial least-squares discriminant analysis model and |log_1.5_(FC)| ≥ 1, according to the variable importance in the projection (VIP) ≥ 1.

## Data accessibility

The data are provided in electronic supplementary material [53].
